# Variability of glucose, insulin, and lipid disturbances in first-episode psychosis: a meta-analysis

**DOI:** 10.1017/S0033291721005213

**Published:** 2023-05

**Authors:** Toby Pillinger, Robert A. McCutcheon, Oliver D. Howes

**Affiliations:** 1 Department of Psychosis Studies, Institute of Psychiatry, Psychology and Neuroscience, King's College of London, London, UK; 2 Psychiatric Imaging Group, MRC London Institute of Medical Sciences, Hammersmith Hospital, Imperial College London, London, UK; 3 Faculty of Medicine, Institute of Clinical Sciences, Imperial College London, London, UK; 4 Department of Psychological Medicine, Institute of Psychiatry, Psychology and Neuroscience, King's College of London, London, UK; 5 H Lundbeck A/s, 3 Abbey View, Everard Close, St Albans AL1 2PS, UK

**Keywords:** cholesterol, diabetes, metabolic, psychosis, schizophrenia, variability

## Abstract

**Background:**

First-episode psychosis (FEP) is associated with metabolic alterations. However, it is not known if there is heterogeneity in these alterations beyond what might be expected due to normal individual differences, indicative of subgroups of patients at greater vulnerability to metabolic dysregulation.

**Methods:**

We employed meta-analysis of variance, indexed using the coefficient of variation ratio (CVR), to compare variability of the following metabolic parameters in antipsychotic naïve FEP and controls: fasting glucose, glucose post-oral glucose tolerance test (OGTT), fasting insulin, insulin resistance, haemoglobin A_1c_ (HbA_1c_), total-cholesterol, low-density lipoprotein (LDL)-cholesterol, high-density lipoprotein (HDL)-cholesterol, and triglycerides. Standardised mean difference in metabolic parameters between groups was also calculated; meta-regression analyses examined physiological/demographic/psychopathological moderators of metabolic change.

**Results:**

Twenty-eight studies were analysed (1716 patients, 1893 controls). Variability of fasting glucose [CVR = 1.32; 95% confidence interval (CI) 1.12–1.55; *p* = 0.001], glucose post-OGTT (CVR = 1.43; 95% CI 1.10–1.87; *p* = 0.008), fasting insulin (CVR = 1.31; 95% CI 1.09–1.58; *p* = 0.01), insulin resistance (CVR = 1.34; 95% CI 1.12–1.60; *p* = 0.001), HbA_1c_ (CVR = 1.18; 95% CI 1.06–1.27; *p* < 0.0001), total-cholesterol (CVR = 1.15; 95% CI 1.01–1.31; *p* = 0.03), LDL-cholesterol (CVR = 1.28; 95% CI 1.09–1.50; *p* = 0.002), and HDL-cholesterol (CVR = 1.15; 95% CI 1.00–1.31; *p* < 0.05), but not triglycerides, was greater in patients than controls. Mean glucose, glucose post-OGTT, fasting insulin, insulin resistance, and triglycerides were greater in patients; mean total-cholesterol and HDL-cholesterol were reduced in patients. Increased symptom severity and female sex were associated with worse metabolic outcomes.

**Conclusions:**

Patients with FEP present with greater variability in metabolic parameters relative to controls, consistent with a subgroup of patients with more severe metabolic changes compared to others. Understanding determinants of metabolic variability could help identify patients at-risk of developing metabolic syndrome. Female sex and severe psychopathology are associated with poorer metabolic outcomes, with implications for metabolic monitoring in clinical practice.

## Introduction

People with psychotic disorders die 15 years earlier than members of the general population (Crump, Winkleby, Sundquist, & Sundquist, 2013). Most of this excess mortality is secondary to physical health conditions, predominantly cardiometabolic disease, highlighting the importance of identifying underlying factors (Osby, Correia, Brandt, Ekbom, & Sparen, 2000; Pillinger et al., 2019b; Pillinger et al., 2019c; Pillinger, D'Ambrosio, McCutcheon, & Howes, 2019a). Although lifestyle and medication play a key role in the metabolic disturbances seen in individuals with psychosis (Pillinger et al., 2020), meta-analyses have observed glucose and lipid alterations from psychosis onset and in the absence of antipsychotic treatment (Greenhalgh et al., 2017; Perry, McIntosh, Weich, Singh, & Rees, 2016; Pillinger et al., 2017a, 2019a; Pillinger, Beck, Stubbs, & Howes, 2017b). Moreover, these alterations persist when patients and controls are matched for factors associated with metabolic function e.g. diet, physical activity, and body mass index (BMI) (Pillinger et al., 2017a, 2017b).

Thus, there is evidence that psychosis is associated with metabolic alterations independent of common risk factors. However, it is not known if psychosis-associated metabolic alterations manifest as a uniform effect (i.e. mean metabolic levels are increased in patients with similar variability of data around the mean for both patients and controls) or, alternatively, if there is heterogeneity in this effect amongst patients beyond what might be expected due to normal individual differences (i.e. mean metabolic levels are increased in patients but also with increased variability of data in patients compared with controls). We set out to address this question by performing a meta-analysis of variability, as previously employed to examine peripheral immune marker and brain structural variability in first-episode psychosis (FEP) (Brugger & Howes, 2017; Pillinger et al., 2019b). If there is heterogeneity in metabolic alterations amongst patients, e.g. because they are seen only in a subgroup of patients, then there will be greater metabolic variability in patients relative to controls. Conversely, if metabolic alterations are a homogenous component of the pathophysiology of psychosis, reduced or similar metabolic variability in patients compared with controls would be predicted, reflecting homogeneity in pathophysiology.

Since publication of previous meta-analyses assessing mean differences in metabolic parameters between FEP and controls (Pillinger et al., 2017a, 2017b), up to eight new case–control studies have been published. Therefore, we also aimed to perform updated meta-analyses of mean differences in metabolic parameters between FEP and controls. The factors underlying metabolic alterations are not clear, and between-study inconsistency has been highlighted as a limitation of previous meta-analyses (Reynolds, 2021), so we additionally investigated factors that might underlie effects and explain inconsistency.

## Methods

A systematic review was performed according to Preferred Reporting Items for Systematic Reviews and Meta-Analyses and Meta-Analysis of Observational Studies in Epidemiology guidelines (Moher, Liberati, Tetzlaff, Altman, & Group, 2009; Stroup et al., 2000) (online Supplementary eAppendix 1), following an *a priori* protocol (PROSPERO: CRD 42021271674). The PubMed, EMBASE, and PsycINFO were searched from inception to August (week 1) 2021 for appropriate articles (online Supplementary eAppendix 2).

### Selection criteria

Inclusion criteria were: (1) patients with diagnoses of schizophrenia, schizoaffective disorder, schizophreniform disorder, schizophrenia spectrum and psychotic disorder not otherwise specified as defined by the Diagnostic and Statistical Manual of Mental Disorders (DSM) or International Statistical Classification of Diseases (ICD); (2) first episode of illness defined as patients within 5-years of first presentation; (3) participants aged above 18 years; (4) antipsychotic-naïve/⩽2 weeks of antipsychotic treatment; (5) a healthy control group; (6) studies assessing plasma fasting glucose, the oral glucose tolerance test (OGTT), fasting insulin, insulin resistance (measured using homoeostatic model assessment for insulin resistance, HOMA-IR), haemoglobin A_1c_ (HbA_1c_), total-cholesterol, low-density lipoprotein (LDL)-cholesterol, high-density lipoprotein (HDL)-cholesterol, and triglyceride levels. Exclusion criteria were: (1) substance/medication-induced psychosis and (2) absent mean and/or variance data.

### Recorded variables

Data were extracted as follows: author, publication year, mean (with standard deviation) measure of metabolic parameter, patient/control number, diagnosis, antipsychotic naïve-status, proportion of males/smokers/non-Caucasians, illness duration, and symptom rating (total Positive and Negative Syndrome Scale (PANSS) scores) (Kay, Fiszbein, & Opler, 1987).

### Statistical analysis

For difference in variability, the natural log of the ratio of estimates of the population standard deviations scaled to mean for each group was calculated, giving the natural logarithm of the ratio of estimates of population coefficients of variation (lnCVR), as previously described (online Supplementary eAppendix 3) (Pillinger et al., 2019b). To aid interpretation, lnCVR effect sizes were transformed to a linear scale; thus, coefficient of variation ratio (CVR) > 1 indicates greater relative variability in patient groups, and CVR < 1 indicates lower relative variability in patient groups.

Standardised mean differences (SMD) in metabolic measurements between patients and controls were also calculated. In expectation of inconsistency across studies, a random-effects model was used; inconsistency was assessed using Cochran *Q* and *I*^2^ statistics (Bowden, Tierney, Copas, & Burdett, 2011; Higgins, Thompson, Deeks, & Altman, 2003). If more than 10 studies were analysed, publication bias was assessed using the Egger test of the intercept (Egger et al., 1997) and represented diagrammatically with funnel plots.

To determine if findings were influenced by confounding, we performed three sensitivity analyses: (1) excluding studies where patients received antipsychotic treatment; (2) excluding studies which included patients other than those with schizophrenia; and (3) excluding studies identified as outliers following leave-one-out diagnostics (Viechtbauer & Cheung, 2010).

Meta-regression analyses were performed to explore the effects of patient age, proportion of males, BMI, proportion of people of non-Caucasian ethnicity, proportion of smokers, illness duration, and total PANSS score on mean metabolic effect size magnitudes. A two-tailed *p* < 0.05 was deemed significant for all analyses.

## Results

### Retrieved studies

The search identified 7975 citations. After exclusion of studies not meeting criteria or reporting overlapping data, 28 studies met inclusion criteria and were analysed. The search process is demonstrated in online Supplementary eAppendix 2, and final studies selected are summarised in online Supplementary eTable 1. The overall sample included 1716 patients and 1893 controls.

### Fasting glucose concentration

**Fig. 1.**
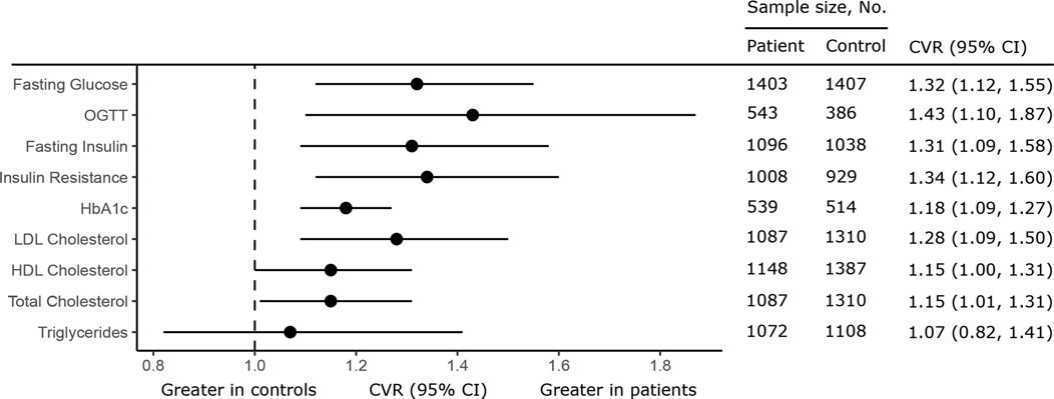
Forest plot showing summary effect sizes for mean-scaled variability of glucose, insulin, and lipid parameters in antipsychotic naïve FEP compared with healthy controls. Each circle shows the summary effect size (CVR); the horizontal line running through each square illustrates the width of the overall 95% CI. The CVR was significantly increased for fasting glucose, glucose after the OGTT, fasting insulin, insulin resistance as measured using HOMA-IR, HbA_1c_, LDL-cholesterol, HDL-cholesterol, and total-cholesterol, indicating greater variability in these metabolic parameters in patients compared with controls. There was no significant difference in CVR for triglyceride levels in patients compared with controls.

**Fig. 2.**
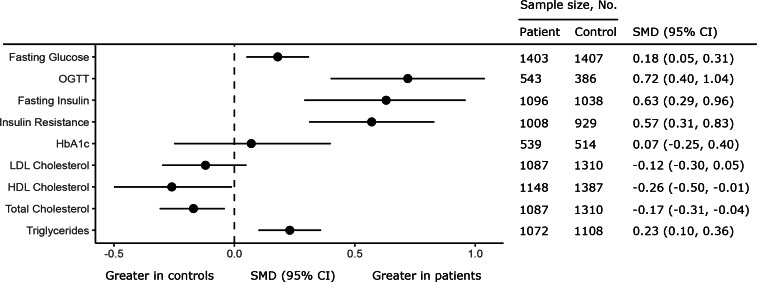
Forest plot showing summary effect sizes for mean differences in glucose, insulin, and lipid parameters in antipsychotic naïve FEP compared with healthy controls. Each circle shows the summary effect size (SMD); the horizontal line running through each square illustrates the width of the overall 95% CI. There was a significant elevation in fasting glucose, glucose levels measured using an OGTT, fasting insulin, insulin resistance as measured using HOMA-IR, and triglyceride levels in patients compared with controls. There was a significant decrease in total and HDL-cholesterol levels in patients compared with controls. There was no significant difference in HbA_1c_ or LDL-cholesterol levels in patients compared with controls.

Fasting glucose was analysed using data from 22 studies comprising 1403 patients and 1407 controls. Fasting glucose was more variable in patients compared with controls [CVR = 1.32; 95% confidence interval (CI) 1.12–1.55; *p* = 0.001] (Fig. 1, online Supplementary eTable 2, eAppendix 4). Mean fasting glucose was also elevated in patients (SMD = 0.18; 95% CI 0.05–0.31; *p* = 0.007) (Fig. 2, online Supplementary eTable 2, eAppendix 4). Between-study inconsistency was not significant (*I*^2^ value = 18.56%; Cochrane *Q* = 26.91, *p* = 0.14). Funnel plot inspection (online Supplementary eAppendix 4) suggested publication bias was not significant (Egger *p* = 0.86). Restricting analyses to antipsychotic naïve patients and those with schizophrenia did not change CVR or SMD outcomes (online Supplementary eTables 3 and 4). Exclusion of two outlying studies (online Supplementary eAppendix 4) for the SMD analysis did not alter results (online Supplementary eTable 5); no CVR outliers were identified. Meta-regression of effect size for mean fasting glucose on patient age, sex, BMI, smoking-status, and illness duration did not yield significant results (online Supplementary eTable 6). However, increased symptom severity was associated with larger fasting glucose effect size (*z* = 2.10, *r* = 0.97, *p* = 0.04) (online Supplementary eAppendix 4). There were insufficient studies to explore the influence of ethnicity on glucose outcomes.

### Glucose concentration after OGTT

Plasma glucose post-OGTT was analysed using data from six studies comprising 543 patients and 386 controls. Glucose concentration post-OGTT was more variable in patients compared with controls (CVR = 1.43; 95% CI 1.10–1.87; *p* = 0.008) (Fig. 1, online Supplementary eTable 2, eAppendix 5). Mean glucose levels post-OGTT were also elevated in patients (SMD = 0.72; 95% CI 0.40–1.04; *p* < 0.0001) (Fig. 2, online Supplementary eTable 2, eAppendix 5). There was between-sample inconsistency (*I*^2^ value = 79.21%; Cochrane *Q* = 22.31, *p* < 0.01). Restricting analyses to antipsychotic naïve patients and those with schizophrenia did not change CVR or SMD outcomes (online Supplementary eTables 3 and 4). Exclusion of a single outlying study (online Supplementary eAppendix 5) for CVR and SMD analyses did not alter results (online Supplementary eTable 5). There were insufficient studies to perform meta-regression analyses.

### Fasting insulin concentration

Fasting insulin concentration was analysed using data from 16 studies comprising 1096 patients and 1038 controls. Fasting insulin was more variable in patients compared with controls (CVR = 1.31; 95% CI 1.09–1.58; *p* = 0.01) (Fig. 1, online Supplementary eTable 2, eAppendix 6). Mean fasting insulin was elevated in patients compared with controls (SMD = 0.63; 95% CI 0.29–0.96; *p* = 0.003) (Fig. 2, online Supplementary eTable 2, eAppendix 6). There was between-sample inconsistency (*I*^2^ value = 91.79%; Cochrane *Q* = 122.77, *p* < 0.01). Funnel plot inspection (online Supplementary eAppendix 6) suggested publication bias was significant (Egger *p* = 0.04). Restricting analyses to antipsychotic naïve patients and those with schizophrenia did not change CVR or SMD outcomes (online Supplementary eTables 3 and 4). Exclusion of a single outlying study (online Supplementary eAppendix 6) for CVR and SMD analyses did not alter results (online Supplementary eTable 5). Meta-regression of effect size for mean fasting insulin on patient age, sex, and BMI did not yield significant results (online Supplementary eTable 6). There were insufficient studies to explore the influence of other moderators.

### Insulin resistance

**Fig. 3.**
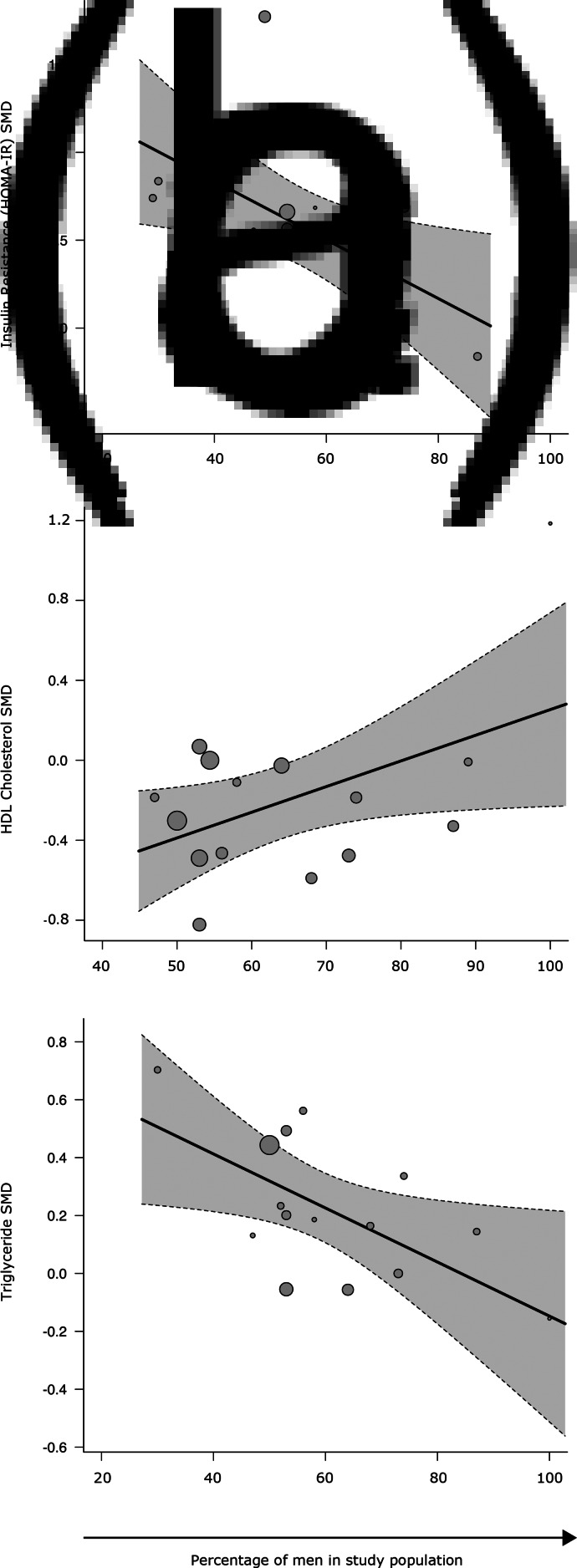
Scatterplots for regression of SMD between patients and controls for metabolic parameters on percentage of men in the patient population. (*a*) Percentage of males in the sample was significantly associated with lower effect size for insulin resistance (as measured using HOMA-IR) effect size (*z* = −2.32, *r* = −0.98, *p* = 0.02); (*b*) percentage of males was significantly associated with lower HDL-cholesterol effect size (*z* = 2.02, *r* = 0.97, *p* = 0.04); and (*c*) percentage of males was significantly associated with lower triglyceride effect size (*z* = −2.18, *r* = −0.98, *p* = 0.03). Each circle represents a study, its size corresponding to the study weight. Single straight line represents the regression coefficient, the curved dotted lines the 95% CI.

Insulin resistance (HOMA-IR) was analysed using data from 13 studies, comprising 1008 patients and 929 controls. HOMA-IR was more variable in patients compared with controls (CVR = 1.34; 95% CI 1.12–1.60; *p* = 0.001) (Fig. 1, online Supplementary eTable 2, eAppendix 7). Mean HOMA-IR was elevated in patients compared with controls (SMD = 0.57; 95% CI 0.31–0.83; *p* < 0.0001) (Fig. 2, online Supplementary eTable 2, eAppendix 7). There was significant between-sample inconsistency (*I*^2^ value = 84.74%; Cochrane *Q* = 67.49, *p* < 0.01). Funnel plot inspection (online Supplementary eAppendix 7) suggested publication bias was not significant (Egger *p* = 0.97). Restricting analyses to antipsychotic naïve patients and those with schizophrenia did not change CVR or SMD outcomes (online Supplementary eTables 3 and 4). Exclusion of an outlying study (online Supplementary eAppendix 7) for the SMD analysis did not alter results (online Supplementary eTable 5); no CVR outliers were identified. Meta-regression of effect size for mean HOMA-IR on patient age and BMI did not yield significant results (online Supplementary eTable 6). However, fewer males in study populations were associated with higher HOMA-IR effect size magnitudes (*z* = −2.32, *r* = −0.98, *p* = 0.02) (Fig. 3). There were insufficient studies to explore the influence of other moderators.

### HbA_1c_ levels

HbA_1c_ levels were analysed using data from five studies comprising 539 patients and 514 controls. HbA_1c_ levels were more variable in patients compared with controls (CVR = 1.18; 95% CI 1.06–1.27; *p* < 0.0001) (Fig. 1, online Supplementary eTable 2, eAppendix 8). However, mean HbA_1c_ levels were not altered in patients compared with controls (SMD = 0.07; 95% CI −0.25 to 0.40; *p* = 0.66) (Fig. 2, online Supplementary eTable 2, eAppendix 8). There was significant between-sample inconsistency (*I*^2^ value = 77.13%; Cochrane *Q* = 23.19, *p* < 0.01). Restricting analyses to antipsychotic naïve patients did not change results for either CVR or SMD outcomes; there were insufficient studies to perform sensitivity analyses restricted to people with schizophrenia (online Supplementary eTables 3 and 4). Exclusion of a single outlying study (online Supplementary eAppendix 8) for CVR and SMD analyses did not alter results (online Supplementary eTable 5). There were insufficient studies to perform meta-regression analyses.

### Total-cholesterol concentration

Total-cholesterol concentration was analysed using data from 14 studies comprising 1087 patients and 1310 controls. Total-cholesterol was more variable in patients compared with controls (CVR = 1.15; 95% CI 1.01–1.31; *p* = 0.03) (Fig. 1, online Supplementary eTable 2, eAppendix 9). Mean total-cholesterol was reduced in patients compared with controls (SMD = −0.17; 95% CI −0.31 to −0.04; *p* = 0.01) (Fig. 2, online Supplementary eTable 2, eAppendix 9). There was significant between-sample inconsistency (*I*^2^ value = 50.55%; Cochrane *Q* = 29.17, *p* < 0.01). Funnel plot inspection (online Supplementary eAppendix 9) suggested publication bias was not significant (Egger *p* = 0.73). Restricting analyses to antipsychotic naïve patients did not change CVR or SMD outcomes (online Supplementary eTable 4). However, restricting analyses to those patients with schizophrenia resulted in no differences in variability or mean difference in total-cholesterol between patients and controls (online Supplementary eTable 3). Exclusion of an outlying study (online Supplementary eAppendix 9) for the CVR analysis did not alter results (online Supplementary eTable 5); no SMD outliers were identified. Meta-regression of effect size for mean total-cholesterol on patient age, sex, and BMI did not yield significant results (online Supplementary eTable 6). There were insufficient studies to explore the influence of other moderators.

### LDL-cholesterol concentration

LDL-cholesterol concentration was analysed using data from 11 studies comprising 1087 patients and 1310 controls. LDL-cholesterol was more variable in patients compared with controls (CVR = 1.28; 95% CI 1.09–1.50; *p* = 0.002) (Fig. 1, online Supplementary eTable 2, eAppendix 10). However, mean LDL-cholesterol was not altered in patients compared with controls (SMD = −0.12; 95% CI −0.30 to 0.05; *p* = 0.17) (Fig. 2, online Supplementary eTable 2, eAppendix 10). There was between-sample inconsistency (*I*^2^ value = 66.11%; Cochrane *Q* = 33.45, *p* < 0.01). Funnel plot inspection (online Supplementary eAppendix 10) suggested publication bias was not significant (Egger *p* = 0.73). Restricting analyses to antipsychotic naïve patients and those with schizophrenia did not change CVR or SMD outcomes (online Supplementary eTables 3 and 4). Exclusion of a single outlying study (online Supplementary eAppendix 10) for CVR and SMD analyses did not alter results (online Supplementary eTable 5). Meta-regression of effect size for mean LDL-cholesterol on patient age, sex, and BMI did not yield significant results (online Supplementary eTable 6). There were insufficient studies to explore the influence of other moderators.

### HDL-cholesterol concentration

HDL-cholesterol concentration was analysed using data from 15 studies comprising 1148 patients and 1387 controls. HDL-cholesterol was more variable in patients compared with controls (CVR = 1.15; 95% CI 1.00–1.31; *p* < 0.05) (Fig. 1, online Supplementary eTable 2, eAppendix 11). Mean HDL-cholesterol was reduced in patients compared with controls (SMD = −0.26; 95% CI −0.50 to −0.01; *p* = 0.04) (Fig. 2, online Supplementary eTable 2, eAppendix 11). There was between-sample inconsistency (*I*^2^ value = 86.79%; Cochrane *Q* = 69.74, *p* < 0.01). Funnel plot inspection (online Supplementary eAppendix 11) did not suggest publication bias (Egger *p* = 0.15). Restricting analyses to antipsychotic naïve patients did not change CVR or SMD outcomes (online Supplementary eTable 3); however, restricting analyses to patients with schizophrenia led to a loss of statistical significance for the SMD (*p* = 0.17), but not the CVR finding (online Supplementary eTable 4). Exclusion of a single outlying study (online Supplementary eAppendix 11) for the SMD analysis did not alter results (online Supplementary eTable 5); no CVR outliers were identified. Meta-regression of effect size for mean HDL-cholesterol on patient age and BMI did not yield significant results (online Supplementary eTable 6). However, more males in study populations were associated with higher HDL-cholesterol effect size magnitudes (*z* = 2.02, *r* = 0.97, *p* = 0.04) (Fig. 3). There were insufficient studies to explore the influence of other moderators.

### Triglyceride concentration

Triglyceride concentration was analysed using data from 15 studies comprising 1072 patients and 1108 controls. There was no difference in triglyceride concentration variability between patients and controls (CVR = 1.07; 95% CI 0.82–1.41; *p* = 0.61) (Fig. 2, online Supplementary eTable 2, eAppendix 12). However, mean triglyceride concentration was increased in patients compared with controls (SMD = 0.23; 95% CI 0.10–0.36; *p* = 0.0006) (Fig. 2, online Supplementary eTable 2, eAppendix 12). There was between-sample inconsistency (*I*^2^ value = 45.89%; Cochrane *Q* = 26.63, *p* = 0.02). Funnel plot inspection (online Supplementary eAppendix 12) suggested publication bias was not significant (Egger *p* = 0.63). Restricting analyses to antipsychotic naïve patients and those with schizophrenia did not change CVR or SMD outcomes (online Supplementary eTables 3 and 4). No outliers were identified. Meta-regression of mean triglyceride effect size on patient age and BMI did not yield significant results (online Supplementary eTable 6). However, fewer males in study population were associated with higher triglyceride effect size magnitudes (*z* = −2.18, *r* = 0.98, *p* = 0.03) (Fig. 3).

## Discussion

Our first main finding is to systematically demonstrate greater variability in virtually all metabolic parameters examined in people with FEP compared with healthy controls, except for triglyceride levels. Antipsychotic treatment does not explain these findings as results remained significant when restricted to samples of antipsychotic naïve patients. Our second main finding is that greater symptom severity is associated with increased fasting glucose levels and female sex is associated with worse metabolic outcomes, namely increased insulin resistance, reduced HDL-cholesterol levels, and raised triglyceride levels. Our third main finding extends the outcomes of previous meta-analyses of mean differences in the field (Pillinger et al., 2017a, 2017b, 2019a) by confirming, with a larger data set, that patients with antipsychotic naïve FEP present with raised fasting glucose levels, glucose levels following the OGTT, fasting insulin levels, insulin resistance and triglyceride levels, and reduced levels of total-cholesterol. In contrast to previously less well-powered meta-analyses, we show that FEP is associated with reductions in HDL-cholesterol levels, and that there is no significant difference between patients and controls for LDL-cholesterol levels. These outcomes were robust to sensitivity analyses that excluded outlying studies.

There is increasing interest in defining psychosis subtypes by peripheral biomarkers, and previous evidence of metabolic differences between patients based on symptom dimensions has been noted (Kirkpatrick, Fernandez-Egea, Garcia-Rizo, & Bernardo, 2009). However, such studies have not addressed whether there is greater metabolic variability in people with psychotic disorders beyond what might be expected due to normal individual differences, with the result that any identified subtypes may be no different from subtypes present in the healthy population. Furthermore, studies that select patients by subtype may result in the sampling of extreme ends of a distribution of similar variance to that seen in controls. Our meta-analysis addresses these issues and extends our understanding of psychosis as a heterogenous disorder by showing, for the first time, altered metabolic variability in FEP.

### Interpretation

One explanation for greater metabolic variability in patient groups may be due to greater variability in adherence to fasting conditions in clinical populations. However, this would not explain increased variability in HbA_1c_ levels seen in patients as this parameter is not affected by fasting; furthermore, in groups where fasting conditions were not uniformly maintained one would expect greater variability in triglyceride levels, which we did not observe. Alternatively, differences in variability could reflect heterogeneity in the biological processes underlying the disorder, indicating that metabolic dysregulation is seen only in some patients, or to varying degrees across patients. Our observation that female sex and more severe symptoms are associated with poorer metabolic outcomes indicates that sex differences and psychopathology may contribute to metabolic alterations in patients. To our knowledge, this is the first study to identify female sex as a potential risk factor for metabolic dysregulation in psychosis. Our observation of a relationship between increased total PANSS score and more severe glucose dysregulation has previously been observed in a large Chinese cohort of patients with antipsychotic naïve FEP (Lang, Li, & Zhang, 2021). However, inspection of the scatter plot for meta-regression of symptom severity on glucose effect size suggests that the association may be driven by one study (online Supplementary eAppendix 4) and results should be interpreted in this context; this highlights the need for future studies to further investigate moderators of metabolic disturbance in psychosis.

### Implications

The results of our meta-analysis build on recent studies exploring metabolic alterations in FEP; beyond emphasising that altered glucose and lipid homoeostasis is present from illness onset, we have identified that there is greater heterogeneity in metabolic parameter levels in patients than that in the healthy population. Meta-analysis of variability has shown that compared to placebo treatment, antipsychotic treatment is associated with greater variability in weight-gain in people with schizophrenia, which may reflect the presence of subgroups of patients who are more vulnerable to antipsychotic-induced metabolic disturbance (Neumeier et al., 2021). It is unclear if heterogeneity in antipsychotic-induced metabolic disturbance is related to heterogeneity in metabolic levels at onset of psychosis; identifying subgroups of patients from illness onset who are more likely to develop metabolic disease following antipsychotic prescription would allow for treatment stratification and precision medicine, and should be a key focus for future research (Pillinger et al., 2020).

Furthermore, understanding the determinants of metabolic variability could provide insight into the biological processes underlying psychosis. For example, a shared role for inflammation in insulin resistance and schizophrenia has been proposed (Perry et al., 2021); however, an inflammatory state may only be seen in a subgroup of patients (Pillinger et al., 2019b), which would explain the metabolic heterogeneity findings documented in the current study. Unfortunately, no studies included in the current meta-analysis provided inflammatory mediator data, precluding a meta-regression analysis exploring the moderating effects of such parameters on metabolic effect size magnitudes. Regardless of the underlying mechanism, recognising that female sex and more severe psychopathology is associated with poorer metabolic outcomes in FEP has implications for metabolic monitoring in clinical practice.

### Strengths and limitations

Compared with previous meta-analyses assessing mean differences (Pillinger et al., 2017a, 2017b), this study has doubled the sample size for several parameters; this increased power provides greater confidence to the finding that antipsychotic naïve FEP is associated with metabolic dysregulation when assessed using mean differences in parameters.

By focusing on antipsychotic-naïve FEP, we limited duration of secondary illness-related factors that influence metabolic parameters. Furthermore, in a previous meta-analysis examining glucose dysregulation in FEP, sensitivity analysis examining studies in which participants were matched for diet and physical activity levels remained significant for raised fasting glucose levels in patients (Pillinger et al., 2017a). However, individuals in the prodromal state already have poorer dietary habits and decreased physical activity compared with age-matched controls, thus we cannot rule out residual confounding in the current meta-analysis (Koivukangas et al., 2010; Stubbs et al., 2016).

Diagnostic heterogeneity is recognised in FEP, which could contribute to variability outcomes. However, our sensitivity analysis of studies that examined only patients with schizophrenia did not change CVR outcomes (except for the total-cholesterol result), so we can be confident that variability results were not entirely the consequence of diagnostic heterogeneity across the psychosis spectrum. Inconsistency between studies was often moderate to high. This could reflect methodological factors, e.g. differences in assay sensitivity. However, the random effects model used is robust to inconsistency, and would not explain our variability findings, because these reflect within-study variation (where methodological factors are the same in both patient and control groups in any given study).

Although correction for multiple testing is not routinely performed in meta-analysis (Cumpston et al., 2019), we recognise that a series of meta-regression analyses were performed without correction; thus, these findings should be considered as hypothesis-generating and future studies are required to test these associations further. Furthermore, several meta-regression analyses were not possible owing to data not being presented, including the influence of ethnicity, illness duration, and proportion of smokers on mean difference and variability outcomes. Future studies should aim to examine these potential moderators.

## Conclusions

Antipsychotic naïve patients with FEP present with both higher levels of glucose and insulin and greater variability in glucose, insulin, and lipid parameters relative to controls, suggesting that subgroups of patients may present with more severe metabolic changes than others. Furthermore, female sex and more severe psychopathology in FEP are associated with metabolic dysregulation.

## Supplementary material

For supplementary material accompanying this paper visit https://doi.org/10.1017/S0033291721005213. click here to view supplementary material
