# Supplementary material for: Variability of glucose, insulin, and lipid disturbances in first-episode psychosis: a meta-analysis
Source: Psychol Med. 2022 Jan 7;53(7):3150–6. doi: 10.1017/S0033291721005213 (PMC10235663; doi:10.1017/S0033291721005213)
Supplement: Supplementary file 1 [file S0033291721005213sup001.docx]

**Variability of glucose, insulin, and lipid disturbances in first-episode psychosis: a meta-analysis**

**SUPPLEMENTARY INFORMATION**

**CONTENTS**

Pages 2-5 eAppendix 1: MOOSE and PRISMA Checklists

Page 6 eAppendix 2: Search process and process of review and exclusion of studies

Page 7 eAppendix 3: Variability calculation

Pages 8-10 eTable 1: Studies included in analysis

Page 11 eTable 2: Results from SMD and CVR meta-analyses

Page 12 eTable 3: Results from sensitivity analysis: patients with schizophrenia only

Page 12 eTable 4: Results from sensitivity analysis: antipsychotic-naïve patients only

Page 13 eTable 5: Results from sensitivity analysis: removal of outliers

Page 14 eTable 6: Results from meta-regression analyses

Pages 15-17 eAppendix 4: Fasting glucose results: SMD and CVR analyses; funnel plot; SMD and CVR outlier diagnostics; scatterplot for regression of standardised mean difference (SMD) between patients and controls for fasting glucose on symptom severity

Page 18-19 eAppendix 5: OGTT results: SMD and CVR analyses; funnel plot; SMD and CVR outlier diagnostics

Pages 20-21 eAppendix 6: Fasting insulin results: SMD and CVR analyses; funnel plot; SMD and CVR outlier diagnostics

Pages 22-23 eAppendix 7: HOMA-IR results: SMD and CVR analyses; funnel plot; SMD and CVR outlier diagnostics

Pages 24-25 eAppendix 8: HbA_1c_ results: SMD and CVR analyses; funnel plot; SMD and CVR outlier diagnostics

Pages 26-27 eAppendix 9: Total cholesterol results: SMD and CVR analyses; funnel plot; SMD and CVR outlier diagnostics

Pages 28-29 eAppendix 10: LDL cholesterol results: SMD and CVR analyses; funnel plot; SMD and CVR outlier diagnostics

Pages 30-31 eAppendix 11: HDL cholesterol results: SMD and CVR analyses; funnel plot; SMD and CVR outlier diagnostics

Pages 32-33 eAppendix 12: Triglyceride results: SMD and CVR analyses; funnel plot; SMD and CVR outlier diagnostics

Pages 34-36 References

**eAppendix 1: MOOSE and PRISMA Checklists**

**MOOSE CHECKLIST**

| **Criteria** | **Brief description of how the criteria were handled in the meta-analysis** |
| --- | --- |
| **Reporting of background should include** | |
| Problem definition | The variability of metabolic and lipid changes in first episode antipsychotic naïve psychosis is unclear. |
| Hypothesis statement | Compared with controls, there will be a difference in variability of metabolic and lipid changes in first episode antipsychotic naïve psychosis |
| Description of study outcomes | Coefficient of Variation Ration (CVR) and Standardised Mean Differences (SMD) in fasting glucose, OGTT, fasting insulin, insulin resistance, HbA1c, total cholesterol, LDL cholesterol, HDL cholesterol, and triglycerides in individuals with first episode schizophrenia (with no or minimal antipsychotic exposure) vs healthy controls were calculated. |
| Type of exposure or intervention used | N/A |
| Type of study designs used | All study designs were included, but only case-control studies were identified. |
| Study Population | Drug naïve (up to 2 weeks’ total antipsychotic lifetime exposure) first episode schizophrenia, and healthy controls. |
| **Reporting of search strategy should include** | |
| Qualifications of searchers | Indicated in the authors list. |
| Search strategy, including time period included in the synthesis and key words | Major electronic databases were searched from inception to August 2021 for studies examining glucose and lipid parameters in individuals with first episode schizophrenia versus healthy controls. Key words and inclusion/exclusion criteria are described in methods section/supplementary information. |
| Databases and registries searched | MEDLINE, EMBASE and PsycINFO were searched. |
| Search Software used, name and version | <https://ovidsp.uk.ovid.com/> |
| Use of hand searching | The search was complemented by hand-searching of meta-analyses and review articles |
| List of citations located and those excluded, including justifications | Detailed in supplementary information |
| Method of addressing articles published in languages other than English | Only articles written in English were considered. |
| Methods of handling abstracts and unpublished studies | We contacted a number of authors for full report of relevant unpublished studies. |
| **Reporting of methods should include** | |
| Description of relevance or appropriateness of studies assembled for assessing the hypothesis to be tested | Inclusion and exclusion criteria are described in the methods section. |
| Rationale for the selection and coding of data | A data extraction sheet was developed (available on request). |
| Assessment of confounding | We conducted sub-group analyse, as described in methods section |
| Assessment of study quality | Bias was assessed using Egger’s test of the intercept and represented diagrammatically with Funnel Plots. |
| Assessment of heterogeneity | The I^2^ value was used to assess heterogeneity. |
| Description of statistical methods in sufficient detail to be replicated | We mentioned the type of analysis we used, and the type of software utilised. |
| Provision of appropriate tables and graphics | Provided both in manuscript and supplementary info. |
| **Reporting of results should include** | |
| Graph summarising individual study estimates and overall estimate | Summary SMD and CVR figures provided in manuscript, individual analyses provided in supplementary information. |
| Table giving descriptive information for each study included | Provided |
| Results of sensitivity testing | Described in results |
| Indication of statistical uncertainty of findings | 95% confidence intervals were presented for all analyses with P values together with I^2^ values for the meta-analyses. |
| **Reporting of discussion should include** | |
| Quantitative assessment of bias | Risk of publication bias was assessed using Egger’s test of the intercept and represented diagrammatically with Funnel Plots |
| Justification for Exclusion | Provided |
| Assessment of quality of included studies | In addition to assessment of bias, discussed in context of limitations in discussion section. |
| **Reporting of conclusions should involve** | |
| Considerations of alternative explanations for observed results | In depth discussion of the potential pathoaetiological mechanisms driving the observations provided. |
| Generalisation of the conclusions | Provided in discussion. |
| Guidelines for future research | Provided in discussion. |
| Disclosure of funding source | Funding statement provided. |

**PRISMA (2009) CHECKLIST**

| Section/Topic | # | Checklist Item | Reported on page # |
| --- | --- | --- | --- |
| TITLE | | | |
| Title | 1 | Identify the report as a systematic review, meta-analysis, or both. | 1 |
| ABSTRACT | | | |
| Structured summary | 2 | Provide a structured summary including, as applicable: background; objectives; data sources; study eligibility criteria, participants, and interventions; study appraisal and synthesis methods; results; limitations; conclusions and implications of key findings; systematic review registration number | 3-4 |
| INTRODUCTION | | | |
| Rationale | 3 | Describe the rationale for the review in the context of what is already known. | 5 |
| Objective | 4 | Provide an explicit statement of questions being addressed with reference to participants, interventions, comparisons, outcomes, and study design (PICOS). | 5 |
| METHODS | | | |
| Protocol and registration | 5 | Indicate if a review protocol exists, if and where it can be accessed (e.g., Web address), and, if available, provide registration information including registration number. | 6 |
| Eligibility criteria | 6 | Specify study characteristics (e.g., PICOS, length of follow-up) and report characteristics (e.g., years considered, language, publication status) used as criteria for eligibility, giving rationale. | 6 |
| Information sources | 7 | Describe all information sources (e.g., databases with dates of coverage, contact with study authors to identify additional studies) in the search and date last searched. | 6 |
| Search | 8 | Present full electronic search strategy for at least one database, including any limits used, such that it could be repeated. | Sup Info |
| Study selection | 9 | State the process for selecting studies (i.e., screening, eligibility, included in systematic review, and, if applicable, included in the meta-analysis). | 6 |
| Data collection process | 10 | Describe method of data extraction from reports (e.g., piloted forms, independently, in duplicate) and any processes for obtaining and confirming data from investigators. | 6 |
| Data items | 11 | List and define all variables for which data were sought (e.g., PICOS, funding sources) and any assumptions and simplifications made. | 6 |
| Risk of bias in individual studies | 12 | Describe methods used for assessing risk of bias of individual studies (including specification of whether this was done at the study or outcome level), and how this information is to be used in any data synthesis. | 7 |
| Summary measures | 13 | State the principal summary measures (e.g., risk ratio, difference in means). | 7 |
| Synthesis of results | 14 | Describe the methods of handling data and combining results of studies, if done, including measures of consistency (e.g., I^2^) for each meta-analysis. | 7 |
| Risk of bias across studies | 15 | Specify any assessment of risk of bias that may affect the cumulative evidence (e.g., publication bias, selective reporting within studies). | 7 |
| Additional analyses | 16 | Describe methods of additional analyses (e.g., sensitivity or subgroup analyses, meta-regression), if done, indicating which were pre-specified. | 7 |
| RESULTS | | | |
| Study selection | 17 | Give numbers of studies screened, assessed for eligibility, and included in the review, with reasons for exclusions at each stage, ideally with a flow diagram. | 8 |
| Study characteristics | 18 | For each study, present characteristics for which data were extracted (e.g., study size, PICOS, follow-up period) and provide the citations. | Sup Info |
| Risk of bias within studies | 19 | Present data on risk of bias of each study and, if available, any outcome level assessment (see item 12). | 8-13 |
| Results of individual studies | 20 | For all outcomes considered (benefits or harms), present, for each study: (a) simple summary data for each intervention group (b) effect estimates and confidence intervals, ideally with a forest plot. | Sup Info |
| Synthesis of results | 21 | Present results of each meta-analysis done, including confidence intervals and measures of consistency. | Fig 1&2 |
| Risk of bias across studies | 22 | Present results of any assessment of risk of bias across studies (see Item 15). | 8-13 |
| Additional analysis | 23 | Give results of additional analyses, if done (e.g., sensitivity or subgroup analyses, meta-regression [see Item 16]). | 8-13 |
| DISCUSSION | | | |
| Summary of evidence | 24 | Summarize the main findings including the strength of evidence for each main outcome; consider their relevance to key groups (e.g., healthcare providers, users, and policy makers). | 13 |
| Limitations | 25 | Discuss limitations at study and outcome level (e.g., risk of bias), and at review-level (e.g., incomplete retrieval of identified research, reporting bias). | 16 |
| Conclusions | 26 | Provide a general interpretation of the results in the context of other evidence, and implications for future research. | 17 |
| FUNDING | | | |
| Funding | 27 | Describe sources of funding for the systematic review and other support (e.g., supply of data); role of funders for the systematic review. | 17 |

**eAppendix 2: Search process and process or review/exclusion of studies**

Searches from previously completed meta-analyses examining the same parameters in the same study population as the current meta-analysis by the same authors were updated.^1, 2^Both of these previous meta-analyses carried out a literature search of The Pubmed, EMBASE, and PsycINFO from inception to 2016. As such, two separate complementary searches of the same databases were performed, with search terms as follows:

Glucose search, updating previous meta-analysis^1^

(schizo* OR psycho*) AND (early OR first) AND (*glucose* or *diabetes* or *type 2* or *prediabetes* or *intolerance* or *oral glucose tolerance test* or *OGTT* or *fasting* or *random* or *insulin* or *insulin resistance* or *hemoglobin A1c or HbA1c* or *homeosta** or *homeostatic model assessment of insulin*)

Lipid search, updating previous meta-analysis^2^

(schizo* OR psycho*) AND (early OR first) AND (metabolic OR lipid OR cholesterol OR HDL OR LDL OR lipoprotein OR triglyceride)

The figure below shows the process of review and exclusion of studies.


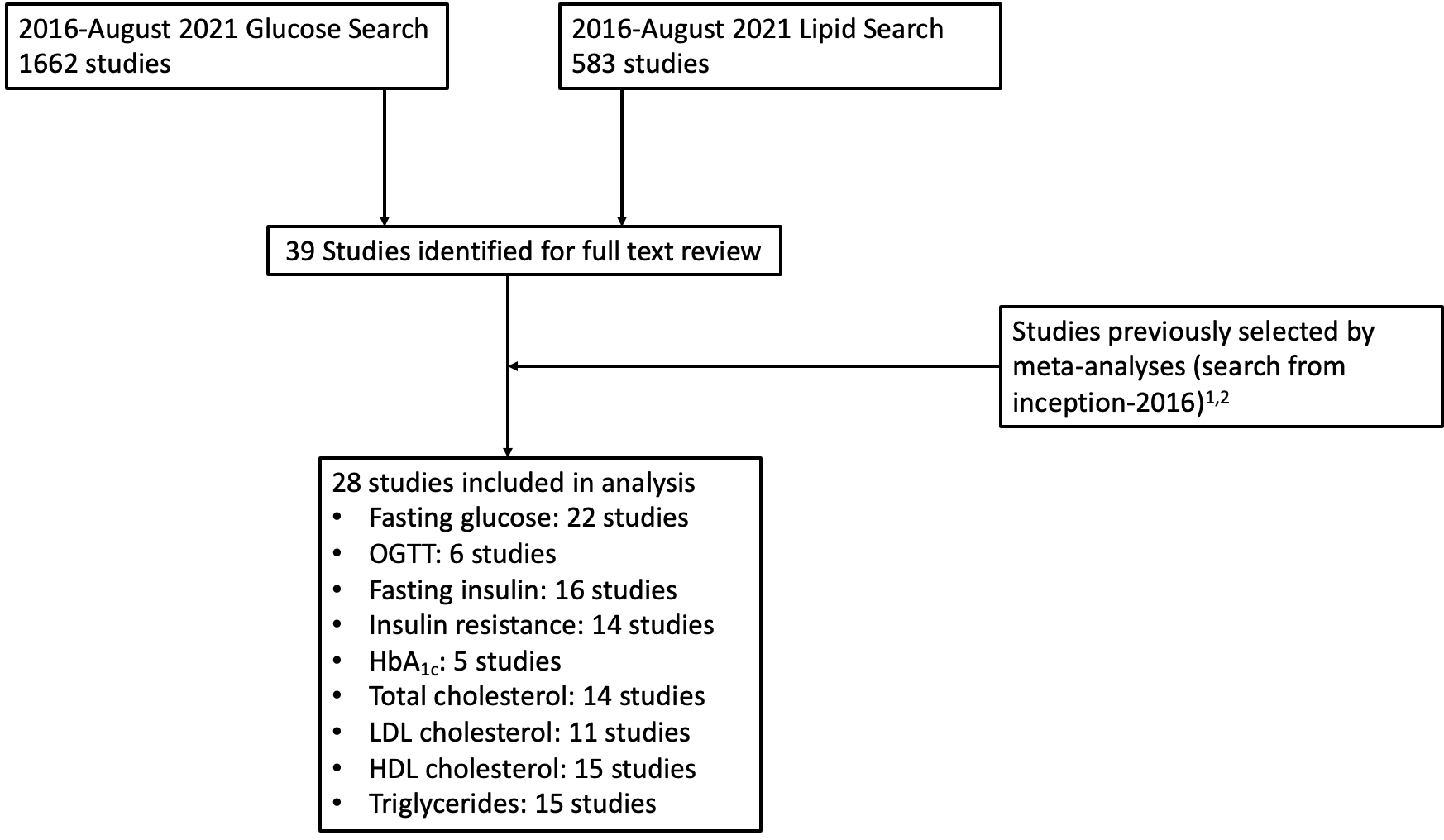


**eAppendix 3: Variability calculation**

Meta-analysis of variability is a technique that compares the variability of measures in one group with another (e.g. patient versus control), indexed by the log variability ratio (lnVR), the natural logarithm of the ratio of standard deviations (SDs) for each group,^3^ as follows:

$$\mathrm{lnVR}=\ln\left( \frac{s_{p}}{s_{c}} \right)+\frac{1}{2(n_{p}-1)}-\frac{1}{2(n_{c}-1)}$$

Where $s_{p}$ and $s_{c}$ are the reported sample SDs, and $n_{p}$ and $n_{c}$ are the sample sizes for patient and control groups, respectively in each case. If alterations in a given biological parameter are only seen in a subgroup of patients, then one would expect greater parameter variability in patients relative to controls. Conversely, if alterations in a given biological process is a core component of the pathophysiology of an illness, reduced parameter variability in patients compared with controls would be predicted.

It is common in biological systems that variance scales with mean, such that larger mean values are associated with greater variance.^4^ Thus, between-group differences in relative variability, although real, may in part reflect between-group differences in mean. To adjust for this, the log coefficient of variation ratio (lnCVR), the natural logarithm of the ratio of unbiased estimates of population coefficients of variation for each group, may be calculated. This relative mean-scaled variability quantifies differences after accounting for differences in mean. The lnCVR is given by the following:

$$\mathrm{lnCVR}=\ln\left( \frac{s_{p}/\bar{x}_{p}}{s_{c}/\bar{x}_{c}} \right)+\frac{1}{2(n_{p}-1)}-\frac{1}{2(n_{c}-1)}$$

Where $\bar{x}_{p}$ and $\bar{x}_{c}$ are the reported means for patient and control groups respectively.

**eTable 1: Studies included in analysis (part 1)**

| Paper | Parameters | Patient number | Control number | Patient Age | % male | Patient BMI |
| --- | --- | --- | --- | --- | --- | --- |
| Arranz 2004^5^ | FG, FI | 50 | 50 | 25.2 | 0.66 | 22 |
| Basoglu 2010^6^ | FG, TC, LDL, HDL, TG | 27 | 22 | 21.2 | 1 | 22 |
| Cai 2012^7^ | FG, FI | 11 | 11 | 27.6 | 0.55 | 21 |
| Chen 2013^8^ | FG, FI, TC, TG | 49 | 30 | 26.8 | 0.29 | 21.6 |
| Chen 2016^9^ | FG, OGTT, FI, HOMA, TC, LDL, HDL, TG | 172 | 31 | 28.7 | 0.52 | 21.8 |
| Chen 2016^10^ | FG, FI, HOMA, TC, TG | 60 | 28 | 28.24 | 0.3 | 21.87 |
| Chen 2018^11^ | FG, FI, OGTT, HOMA, TC, LDL, HDL, TG | 100 | 118 | 25.51 | 0.53 | 20.73 |
| Cohn 2006^12^ | FG, FI | 10 | 10 | 26.6 | 0.89 | 23.7 |
| Dasgupta 2010^13^ | FG, HOMA, TC, LDL, HDL, TG | 30 | 25 | 32.53 | 0.47 | 20.95 |
| Enez Darcin 2015^14^ | FG, FI, HOMA, HDL, TG | 40 | 70 | 34.6 | 0.73 | 24.3 |
| Fernandez Egea 2009^15^ | FG, FI, HOMA, OGTT, HbA1c | 50 | 50 | 29.4 | 0.7 | 22.9 |
| Garcia Rizo 2016^16^ | FG, FI, OGTT | 84 | 98 | 27.28 | 0.64 | 22.13 |
| Kavzoglu 2013^17^ | FG, TC, LDL, HDL, TG | 50 | 50 | 30.14 | 0.5 | NAN |
| Kirkpatrick 2010^18^ | TC, LDL, HDL, TG | 76 | 76 | 27.1 | 0.64 | 22.3 |
| Lang 2021^19^ | FG, FI, HOMA, HbA1c, TC, LDL, HDL, TG | 373 | 350 | 34.48 | 0.5 | 23.73 |
| Misiak 2016^20^ | FG, TC, LDL, HDL, TG | 24 | 146 | 27.2 | 0.56 | 23 |
| Petrikis 2015^21^ | FG, Fi, HOMA, HbA1c | 40 | 40 | 32.45 | 0.68 | 22.88 |
| Phutane 2011^22^ | TC, HDL | 14 | 145 | 22.5 | 0.89 | 25.8 |
| Ryan 2003^23^ | FG, FI, HOMA, TC, LDL, HDL, TG | 26 | 26 | 33.6 | 0.58 | 24.5 |
| Saddichha 2008^24^ | FG, TG, HDL | 99 | 51 | 26 | 0.53 | NAN |
| Sengupta 2008^25^ | FG, FI, HOMA, HBA1c, TC, LDL, HDL, TG | 38 | 36 | 25.4 | 0.87 | 22.8 |
| Spelman 2007^26^ | FG, OGTT, FiI, HOMA, HbA1c, TC, LDL, HDL, TG | 38 | 38 | 25.2 | 0.74 | 22.8 |
| Sun 2016^27^ | FI | 13 | 15 | 22.54 | 1 | NAN |
| Tao 2020^28^ | FG, FI, HOMA | 90 | 70 | 21.5 | 0.49 | 21.5 |
| Verma 2009^29^ | TC, LDL, HDL | 160 | 200 | 30 | 0.544 | 21.2 |
| Venkatasubramanian 2007^30^ | FG, FI, HOMA | 44 | 44 | 33 | 0.52 | NAN |
| Wani 2015^31^ | FG, OGTT | 50 | 50 | 25.4 | 0.64 | NAN |
| Wu 2013^32^ | FG, FI, HOMA, TC, HDL, LDL, TG | 70 | 44 | 24.49 | 0.53 | 19.63 |

**eTable 1: Studies included in analysis (part 2)**

| Paper | All naïve? | All DSM scz? | % non-caucasian | Illness duration (months) | % smokers | PANSS total |
| --- | --- | --- | --- | --- | --- | --- |
| Arranz 2004 | yes | yes | NAN | NAN | NAN | NAN |
| Basoglu 2010 | yes | yes | NAN | NAN | 0.65 | 95.2 |
| Cai 2012 | yes | yes | NAN | 9.6 |  | 55.6 |
| Chen 2013 | no | yes | 1 | NAN | 0.14 | 84.33 |
| Chen 2016 | yes | yes | 1 | 23.4 | 0.081 | 86.61 |
| Chen 2016 | no | yes | 1 | 15.5 | 0.12 | 85.31 |
| Chen 2018 | yes | yes | NAN | NAN | NAN | NAN |
| Cohn 2006 | yes | no | 0.44 | NAN | 0.44 | NAN |
| Dasgupta 2010 | yes | yes | NAN | NAN | NAN | NAN |
| Enez Darcin 2015 | yes | yes | NAN | 20.4 | 0.51 | NAN |
| Fernandez Egea 2009 | yes | no | 0.02 | NAN | NAN | NAN |
| Garcia Rizo 2016 | no | no | NAN | NAN | NAN | NAN |
| Kavzoglu 2013 | no | yes | NAN | NAN | NAN | NAN |
| Kirkpatrick 2010 | no | no | NAN | NAN | NAN | NAN |
| Lang 2021 | yes | yes | 1 | 17 | 0.25 | 116 |
| Misiak 2016 | yes | yes | NAN | 26.2 | NAN | 77.7 |
| Petrikis 2015 | yes | no | NAN | 10.72 | 0.33 | 77.1 |
| Phutane 2011 | yes | no | 0.69 | NAN | 0.46 | NAN |
| Ryan 2003 | yes | yes | 0 | NAN | NAN | NAN |
| Saddichha 2008 | yes | yes | NAN | 20.5 |  | NAN |
| Sengupta 2008 | no | no | 15.8 | 6.25 | 0.79 | NAN |
| Spelman 2007 | yes | yes | 0 | NAN | NAN | NAN |
| Sun 2016 | yes | yes | 1 | 12.08 |  | 108.23 |
| Tao 2020 | yes | yes | NAN | 5.9 | 0.06 | 84.2 |
| Verma 2009 | no | no | 1 | 36.7 | NAN | NAN |
| Venkatasubramanian 2007 | yes | yes | NAN | 39.2 | NAN | NAN |
| Wani 2015 | yes | yes | NAN | NAN | NAN | NAN |
| Wu 2013 | yes | yes | 1 | 6 | NAN | 92.04 |

**eTable 2: Results from SMD and CVR meta-analyses**

| **Parameter** | **Study N** | **Patient/Control N** | **Result** | **Interpretation** |
| --- | --- | --- | --- | --- |
| **Fasting Glucose** | 22 | 1403/1407 | SMD= 0.18 (95%CI: 0.05-0.31; p=0.007) | ↑ fasting glucose in FEP |
|  |  |  | CVR= 1.32 (95%CI: 1.12-1.55; p=0.001) | ↑ glucose variability in FEP |
| **OGTT** | 6 | 543/386 | SMD= 0.72 (95%CI: 0.40-1.04; p<0.0001) | ↑ glucose post-OGTT in FEP |
|  |  |  | CVR= 1.43 (95%CI: 1.10-1.87; p=0.008) | ↑ glucose variability post-OGTT in FEP |
| **Fasting insulin** | 16 | 1096/1038 | SMD= 0.63 (95%CI: 0.29-0.96; p=0.003) | ↑ fasting insulin in FEP |
|  |  |  | CVR= 1.31 (95%CI: 1.09-1.58; p=0.01) | ↑ insulin variability in FEP |
| **HOMA-IR** | 13 | 1008/929 | SMD= 0.57 (95%CI: 0.31-0.83; p<0.0001) | ↑ insulin resistance in FEP |
|  |  |  | CVR= 1.34 (95%CI: 1.12-1.60; p=0.001) | ↑ insulin resistance variability in FEP |
| **HbA1c** | 5 | 539/514 | SMD= 0.07 (95%CI: -0.25-0.40; p=0.66) | No difference in HbA1c btw FEP&HC |
|  |  |  | CVR= 1.18 (95%CI: 1.06-1.27; p<0.0001) | ↑ HbA1c variability in FEP |
| **Total cholesterol** | 14 | 1087/1310 | SMD= -0.17 (95%CI: -0.31 - -0.04; p=0.01) | ↓ total cholesterol in FEP |
|  |  |  | CVR= 1.15 (95%CI: 1.01- 1.31; p=0.03) | ↑ total cholesterol variability in FEP |
| **LDL Cholesterol** | 11 | 955/1081 | SMD= -0.12 (95%CI: -0.30-0.05; p=0.17) | No difference in LDL chol btw FEP&HC |
|  |  |  | CVR= 1.28 (95%CI: 1.09-1.50; p=0.002) | ↑ LDL cholesterol variability in FEP |
| **HDL Cholesterol** | 15 | 1148/1387 | SMD= -0.26 (95%CI: -0.50 - -0.01; p=0.04) | ↓ HDL cholesterol in FEP |
|  |  |  | CVR= 1.15 (95%CI: 1.00-1.31; p<0.05) | ↑ HDL cholesterol variability in FEP |
| **Triglycerides** | 15 | 1072/1108 | SMD= 0.23 (95%CI: 0.10-0.36; p = 0.0006) | ↑ triglycerides in FEP |
|  |  |  | CVR= 1.07 (95%CI: 0.82-1.41; p=0.61) | No diff in TG variability btw FEP&HC |

**eTable 3: Results from sensitivity analysis: patients with schizophrenia only**

| **Parameter** | **Study N** | **Result** |
| --- | --- | --- |
| **Fasting Glucose** | 18 | SMD= 0.21 (95%CI: 0.07-0.35; p=0.004) |
|  |  | CVR= 1.28 (95%CI: 1.06-1.55; p=0.01) |
| **OGTT** | 5 | SMD= 0.68 (95%CI: 0.29-1.07; p=0.0006) |
|  |  | CVR= 1.53 (95%CI: 1.14-2.04; p=0.004) |
| **Fasting insulin** | 13 | SMD= 0.73 (95%CI: 0.33-1.12; p=0.0003) |
|  |  | CVR= 1.26 (95%CI: 1.12-1.41; p=0.0001) |
| **HOMA-IR** | 11 | SMD= 0.68 (95%CI: 0.42-0.94; p<0.0001) |
|  |  | CVR= 1.37 (95%CI: 1.17-1.61; p<0.0001) |
| **HbA1c** | 2 | n/a |
|  |  | n/a |
| **Total cholesterol** | 9 | SMD= -0.15 (95%CI: -0.34-0.04; p=0.12) |
|  |  | CVR= 1.21 (95%CI: 1.00-1.48; p=0.05) |
| **LDL Cholesterol** | 8 | SMD= -0.05 (95%CI: -0.25-0.15; p=0.65) |
|  |  | CVR= 1.36 (95%CI: 1.11-1.65; p=0.003) |
| **HDL Cholesterol** | 10 | SMD= -0.26 (95%CI: -0.63-0.11; p=0.17) |
|  |  | CVR= 1.21 (95%CI: 1.03-1.42; p=0.02) |
| **Triglycerides** | 12 | SMD= 0.27 (95%CI: 0.12-0.42; p=0.0003) |
|  |  | CVR= 1.12 (95%CI: 0.80-1.58; p=0.50 |

**eTable 4: Results from sensitivity analysis: antipsychotic-naïve patients only**

| **Parameter** | **Study N** | **Result** |
| --- | --- | --- |
| **Fasting Glucose** | 17 | SMD= 0.26 (95%CI: 0.11-0.40; p=0.0004) |
|  |  | CVR= 1.34 (95%CI: 1.08-1.66; p=0.008) |
| **OGTT** | 5 | SMD= 0.68 (95%CI: 0.29-1.07; p=0.0006) |
|  |  | CVR= 1.53 (95%CI: 1.14-2.04; p=0.004) |
| **Fasting insulin** | 12 | SMD= 0.71 (95%CI: 0.27-1.15; p=0.002) |
|  |  | CVR= 1.28 (95%CI: 1.11-1.46; p=0.0005) |
| **HOMA-IR** | 10 | SMD= 0.60 (95%CI: 0.30-0.90; p<0.0001) |
|  |  | CVR= 1.43 (95%CI: 1.20-1.71; p<0.0001) |
| **HbA1c** | 4 | SMD= 0.08 (95%CI: -0.32-0.49; p=0.69) |
|  |  | CVR= 1.17 (95%CI: 1.08-1.27; p=0.0001) |
| **Total cholesterol** | 14 | SMD= -0.17 (95%CI: -0.31- -0.04; p=0.01) |
|  |  | CVR= 1.15 (95%CI: 1.01-1.31; p=0.03) |
| **LDL Cholesterol** | 8 | SMD= -0.05 (95%CI: -0.25-0.15; p=0.65) |
|  |  | CVR= 1.36 (95%CI: 1.11-1.65; p=0.003) |
| **HDL Cholesterol** | 12 | SMD= -0.28 (95%CI: -0.60-0.03; p=0.08) |
|  |  | CVR= 1.14 (95%CI: 0.98-1.33; p=0.09) |
| **Triglycerides** | 12 | SMD= 0.24 (95%CI: 0.10-0.38; p=0.0009) |
|  |  | CVR= 1.10 (95%CI: 0.79-1.53; p=0.59) |

**eTable 5: Results from sensitivity analysis: removal of outliers**

| **Parameter** | **Study N** | **Result** |
| --- | --- | --- |
| **Fasting Glucose** | 21 | SMD= 0.13 (95%CI: 0.04-0.23; p=0.006) |
|  |  | CVR= n/a |
| **OGTT** | 5 | SMD= 0.84 (95%CI: 0.62-1.07; p<0.0001) |
|  |  | CVR= 1.27 (95%CI: 1.07-1.50; p=0.006) |
| **Fasting insulin** | 15 | SMD= 0.50 (95%CI: 0.23-0.80; p=0.0004) |
|  |  | CVR= 1.22 (95%CI: 1.06-1.40; p=0.007) |
| **HOMA-IR** | 12 | SMD= 0.46 (95%CI: 0.30-0.63; p<0.0001) |
|  |  | CVR= n/a |
| **HbA1c** | 4 | SMD= -0.08 (95%CI: -0.34-0.18; p=0.54) |
|  |  | CVR= 1.23 (95%CI: 1.08-1.42; p=0.003) |
| **Total cholesterol** | 13 | SMD= n/a |
|  |  | CVR= 1.10 (95%CI: 1.04-1.17; p=0.001) |
| **LDL Cholesterol** | 10 | SMD= -0.06 (95%CI: -0.22 – 0.10; p=0.48) |
|  |  | CVR= 1.21 (95%CI: 1.07-1.37; p=0.002) |
| **HDL Cholesterol** | 14 | SMD= -0.35 (95%CI: -0.52 - -0.17; p=0.0001) |
|  |  | CVR= n/a |
| **Triglycerides** | n/a | SMD= n/a |
|  |  | CVR= n/a |

**eTable 6: Results from SMD meta-regression analyses**

| **Parameter** | **Meta-regression** | | | | | | |
| --- | --- | --- | --- | --- | --- | --- | --- |
|  | **Age** | **Sex** | **BMI** | **Smoking** | **Ethnicity** | **Illness duration** | **Symptom severity** |
| **Fasting glucose** | Z= -0.38  P= 0.70 | Z= 1.00  P= 0.32 | Z= 0.18  P= 0.86 | Z= 0.36  P= 0.62 | n/a | Z= 0.61  P= 0.54 | **Z= 2.10**  **P= 0.04** |
| **OGTT** | n/a | n/a | n/a | n/a | n/a | n/a | n/a |
| **Fasting insulin** | Z= -1.48  P= 0.14 | Z= -0.24  P= 0.81 | Z= -1.08  P= 0.28 | n/a | n/a | n/a | n/a |
| **HOMA-IR** | Z= -1.79  P= 0.07 | **Z= -2.32**  **P= 0.02** | Z= -1.22  P= 0.22 | n/a | n/a | n/a | n/a |
| **HbA1c** | n/a | n/a | n/a | n/a | n/a | n/a | n/a |
| **Total cholesterol** | Z= 0.10  P= 0.92 | Z= -0.56  P= 0.58 | Z= 1.30  P= 0.19 | n/a | n/a | n/a | n/a |
| **LDL cholesterol** | Z= 1.05  P= 0.21 | Z= 0.94  P= 0.35 | Z= -0.10  P= 0.92 | n/a | n/a | n/a | n/a |
| **HDL cholesterol** | Z= 0.87  P= 0.39 | **Z= 2.02**  **P= 0.04** | Z= 0.52  P= 0.60 | n/a | n/a | n/a | n/a |
| **Triglycerides** | Z= 0.63  P= 0.53 | **Z= -2.18**  **P= 0.03** | Z= 0.78  P= 0.44 | n/a | n/a | n/a | n/a |

**eAppendix 4: Fasting glucose results**

**SMD forest plot (fasting glucose)**

**
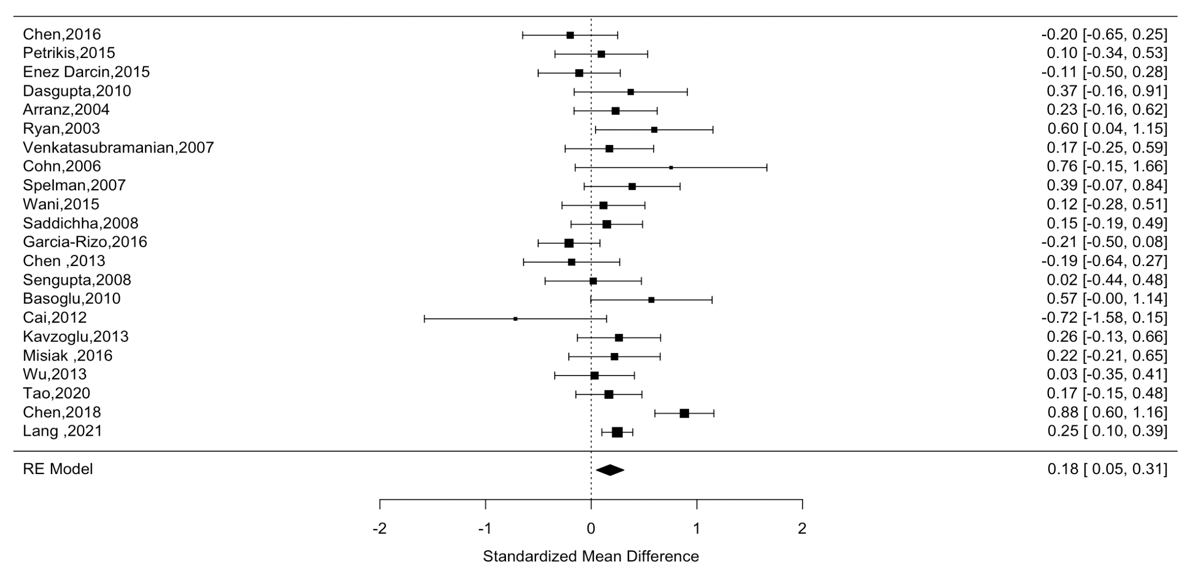
**

**CVR forest plot (fasting glucose)**

**
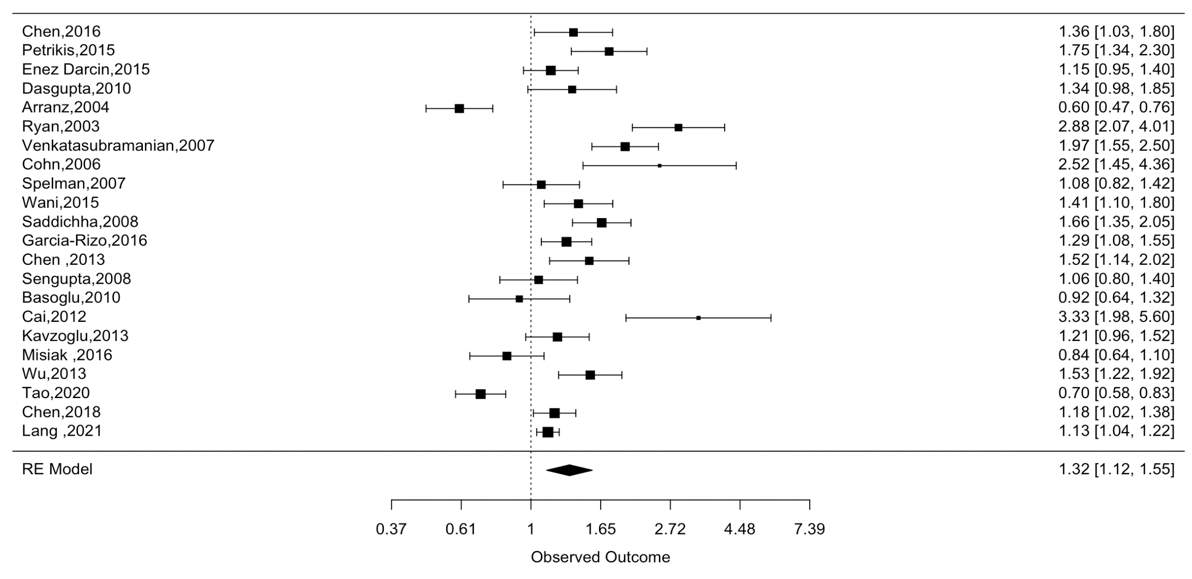
**

**Funnel plot (fasting glucose)**

**
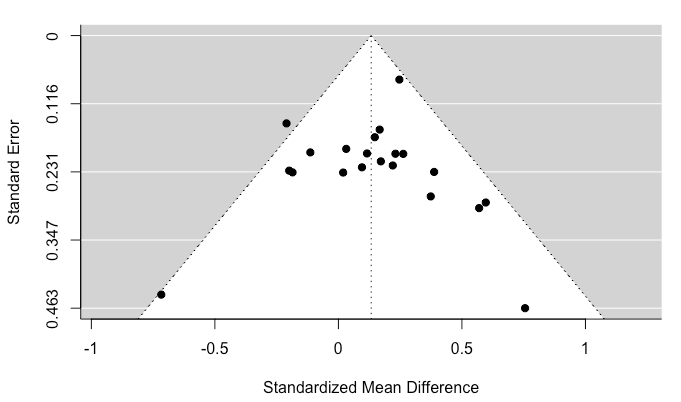
**

**SMD outlier analysis (fasting glucose)**

**
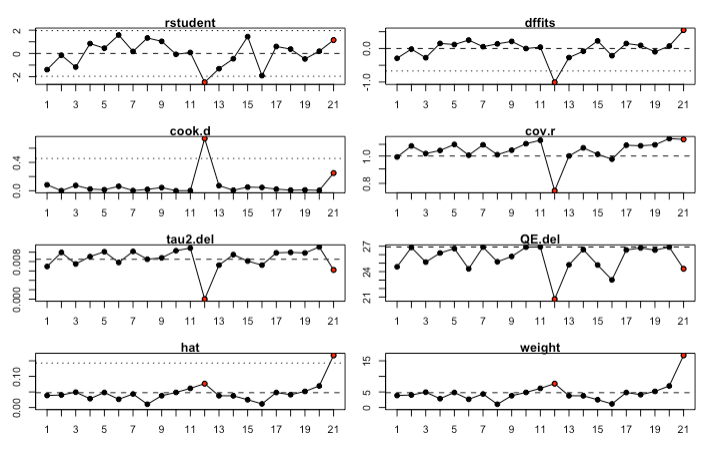
**

Studies highlighted in red are identified as potential outliers. Studies 12 and 21 correspond to Chen et al., 2018^11^ and Garcia-Rizo et al., 2016.^16^

**CVR outlier analysis (fasting glucose)**

**
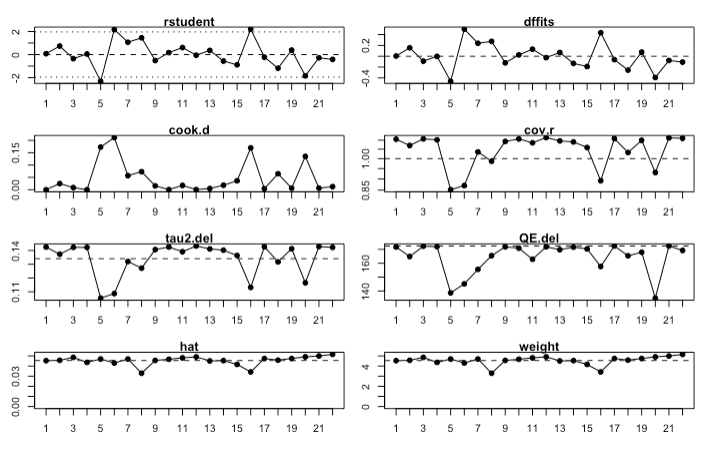
**

No outliers identified


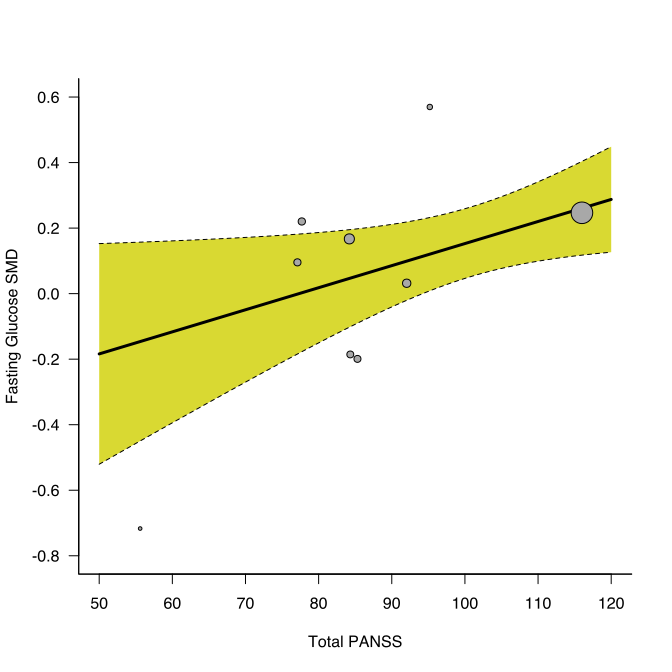


**Scatterplot for regression of standardised mean difference (SMD) between patients and controls for fasting glucose on symptom severity.** Greater severity of symptoms as assessed using Positive and Negative Syndrome Scale (PANSS) total scores was significantly associated with larger elevation in fasting glucose levels in patients relative to controls (z=2.10, r=0.97, p=0.04). Each circle represents a study, its size corresponding to the study weight. Single straight line represents the regression coefficient, the curved dotted lines the 95% confidence interval.

**eAppendix 5: OGTT results**

**SMD forest plot (OGTT)**

**
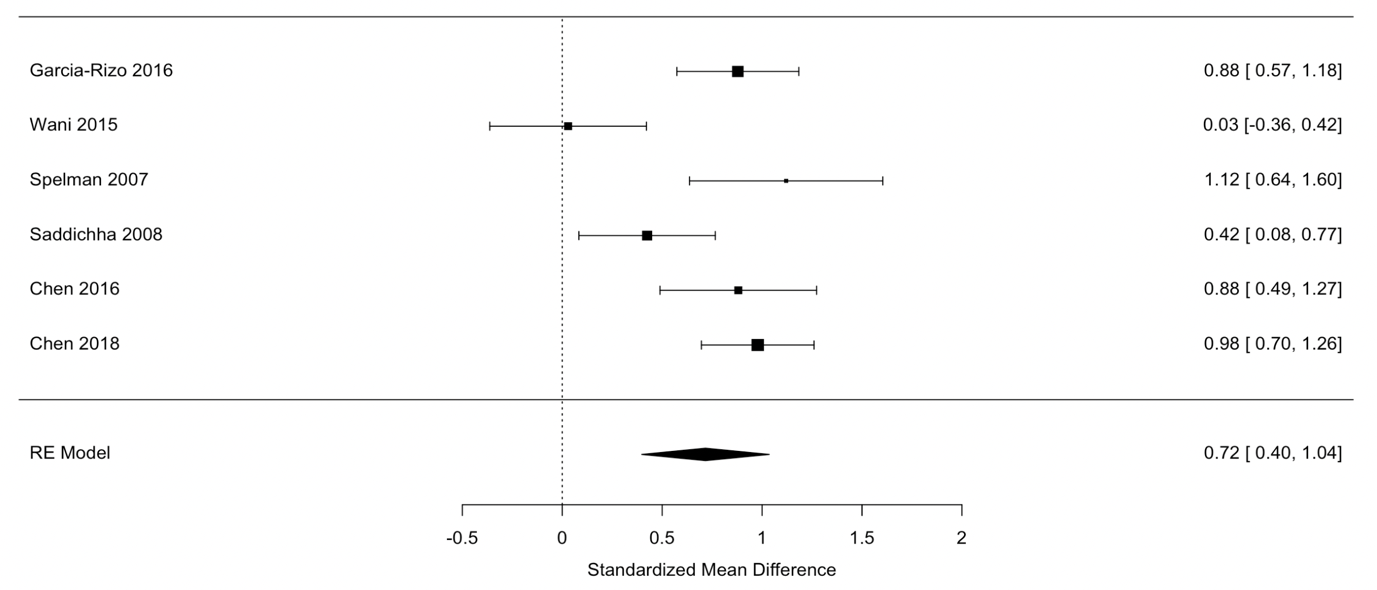
**

**CVR forest plot (OGTT)**

**
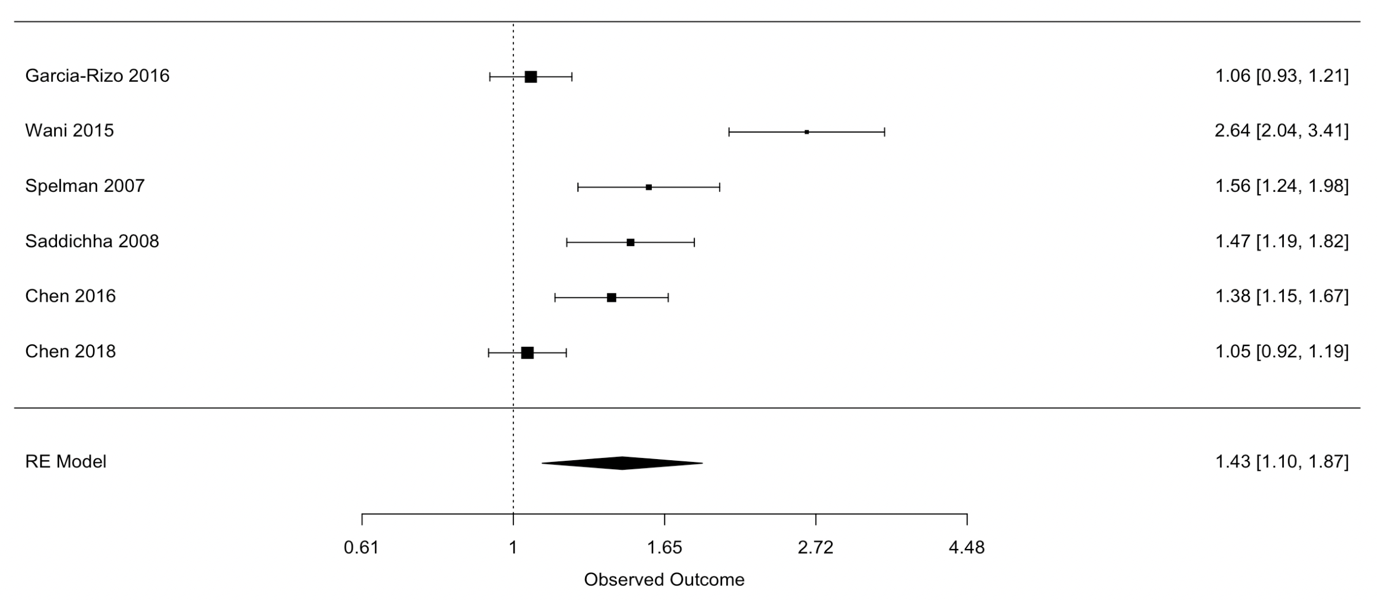
**

**SMD outlier analysis (OGTT)**

**
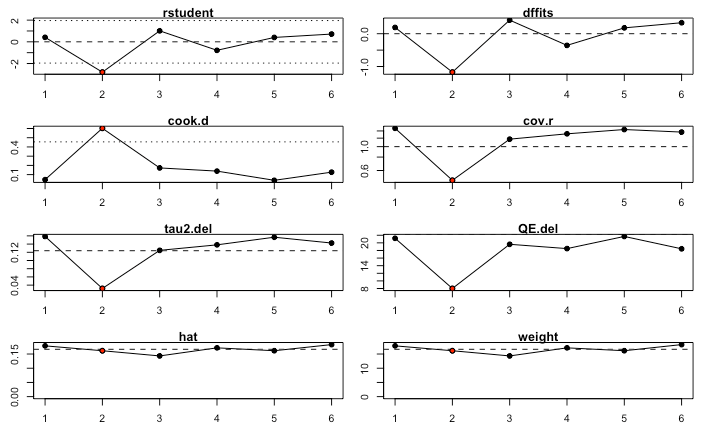
**

The study highlighted in red is identified as a potential outlier. Study 2 corresponds to Wani et al., 2015.^31^

**CVR outlier analysis (OGTT)**

**
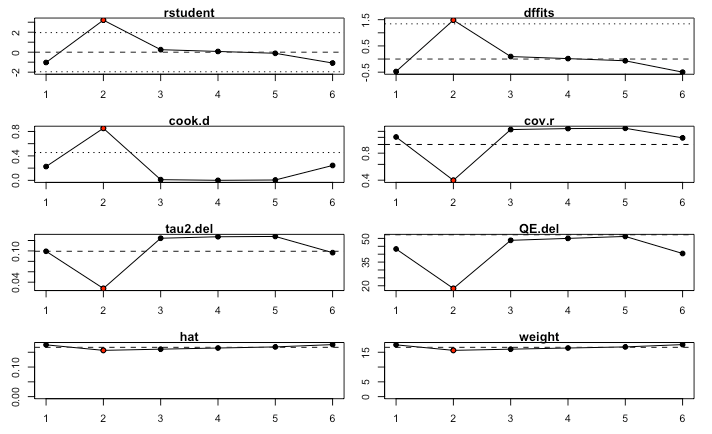
**

The study highlighted in red is identified as a potential outlier. Study 2 corresponds to Wani et al., 2015.^31^

**eAppendix 6: Fasting insulin results**

**SMD forest plot (fasting insulin)**

**
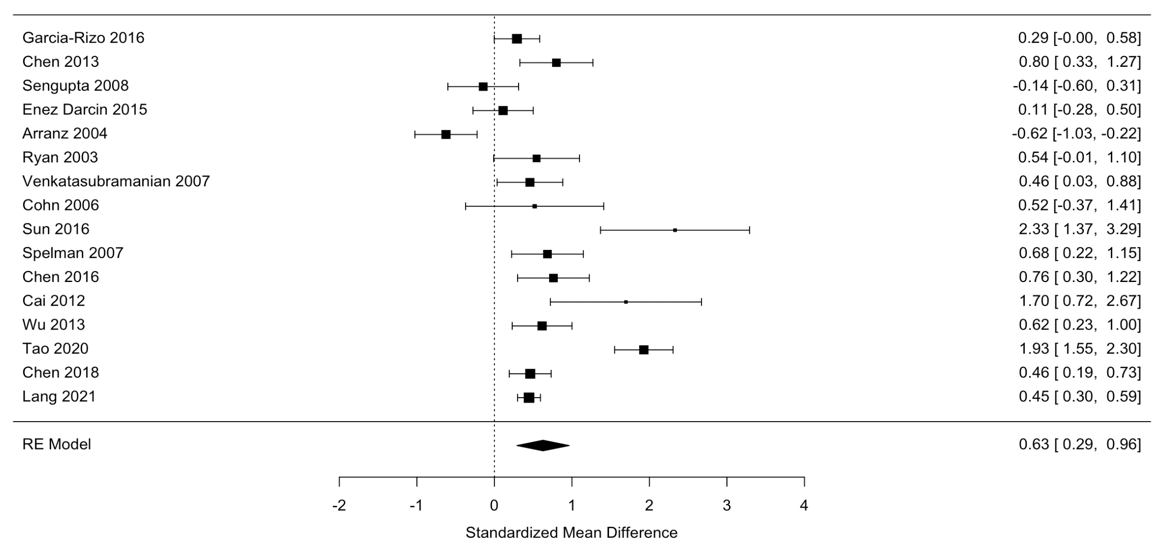
**

**CVR forest plot (fasting insulin)**

**
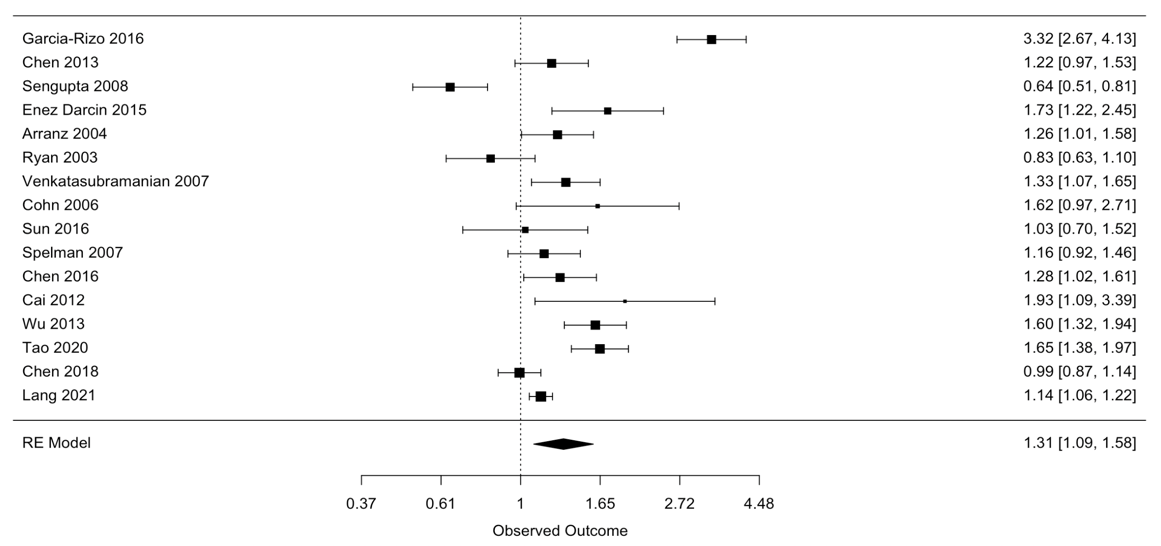
**

**Funnel plot (fasting insulin)**

**
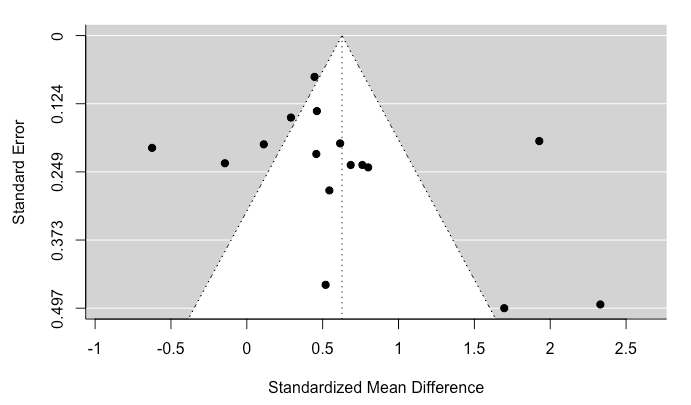
**

**SMD outlier analysis (fasting insulin)**

**
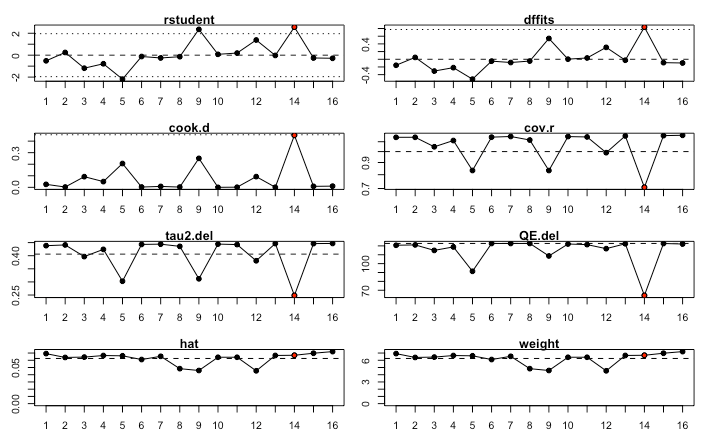
**

The study highlighted in red is identified as a potential outlier. Study 14 corresponds to Tao et al., 2020.^28^

**CVR outlier analysis (fasting insulin)**

**
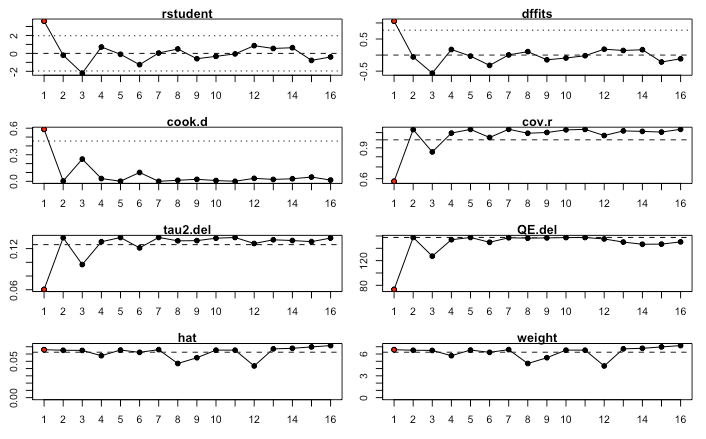
**

The study highlighted in red is identified as a potential outlier. Study 1 corresponds to Garcia Rizo et al., 2016.^16^

**eAppendix 7: HOMA-IR results**

**SMD forest plot (HOMA-IR)**

**
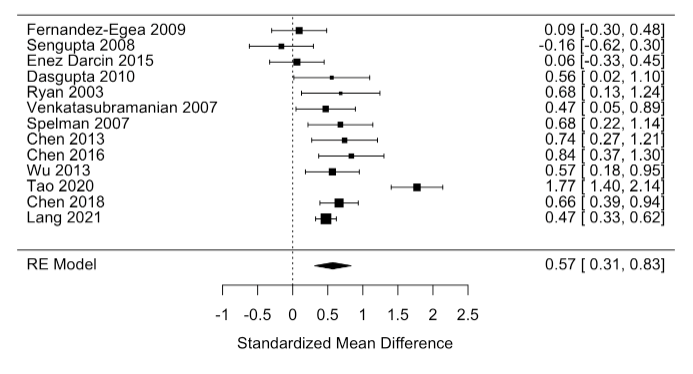
**

**CVR forest plot (HOMA-IR)**

**
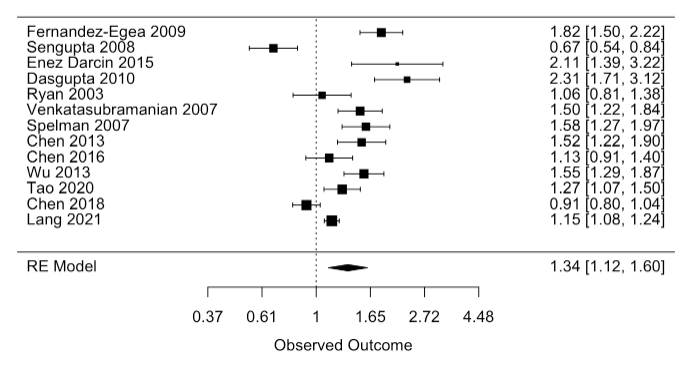
**

**Funnel plot (HOMA-IR)**

**
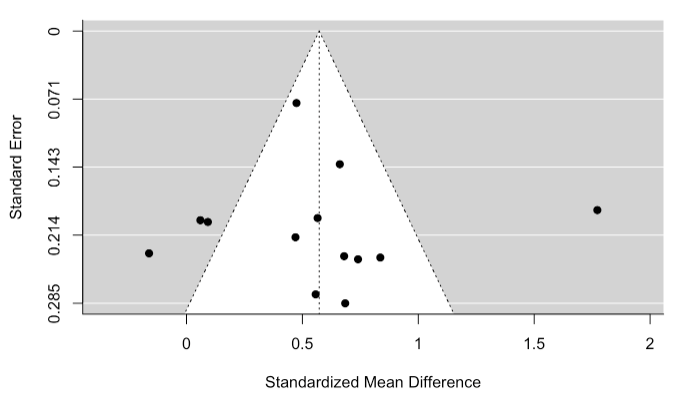
**

**SMD outlier analysis (HOMA-IR)**


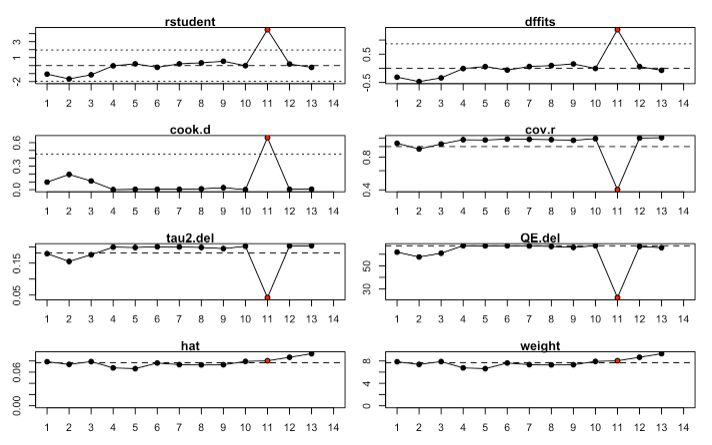


The study highlighted in red is identified as a potential outlier. Study 11 corresponds to Tao et al., 2020.^28^

**CVR outlier analysis (HOMA-IR)**

**
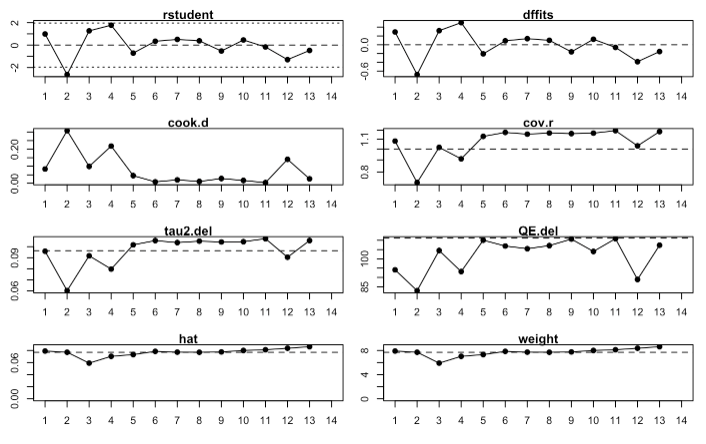
**

No outliers identified.

**eAppendix 8: HbA_1c_ results**

**SMD forest plot (HbA_1c_)**

**
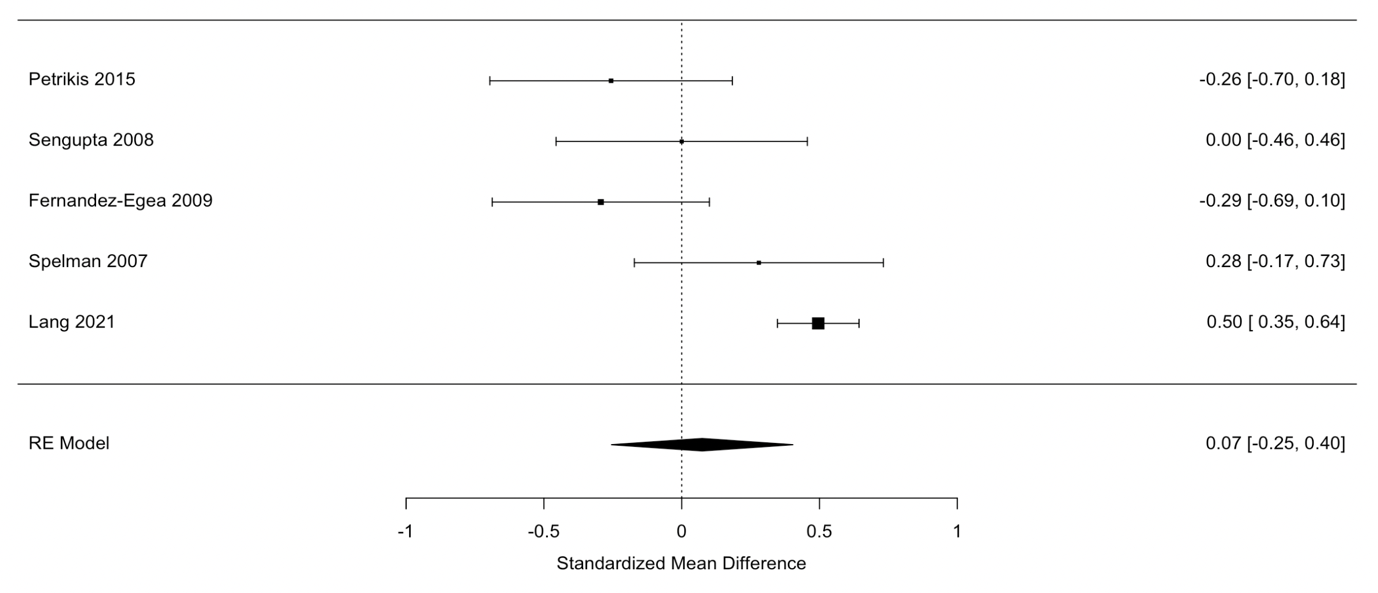
**

**CVR forest plot (HbA_1c_)**

**
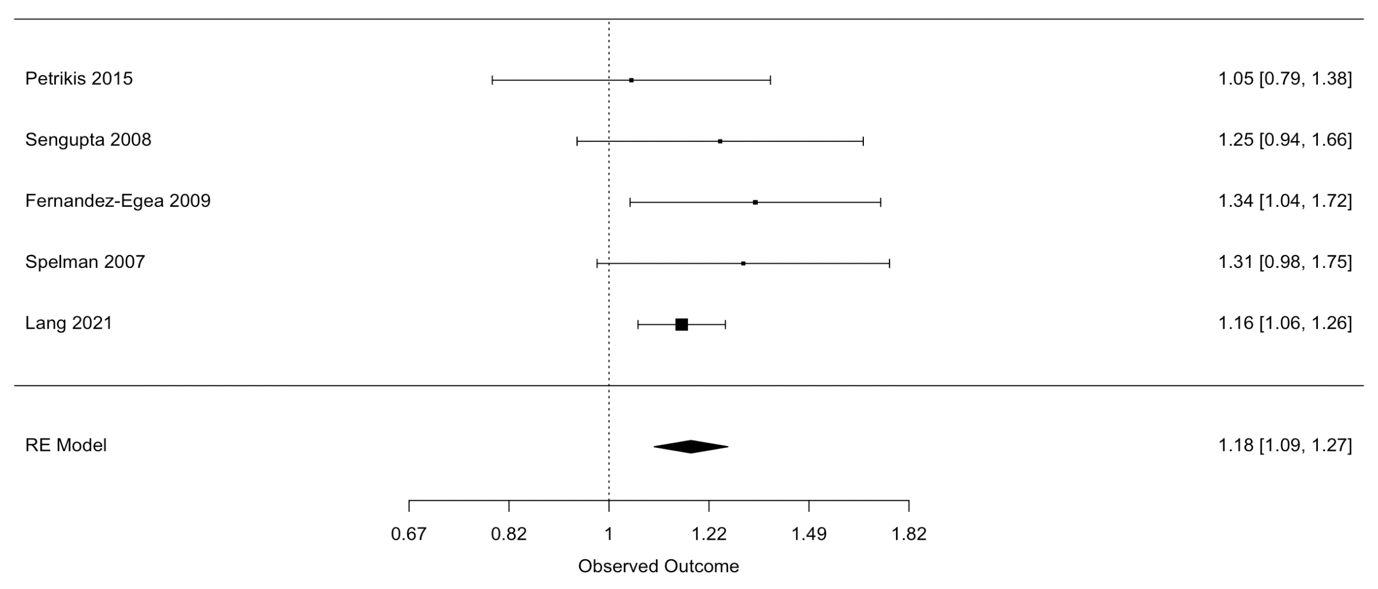
**

**SMD outlier analysis (HbA_1c_)**


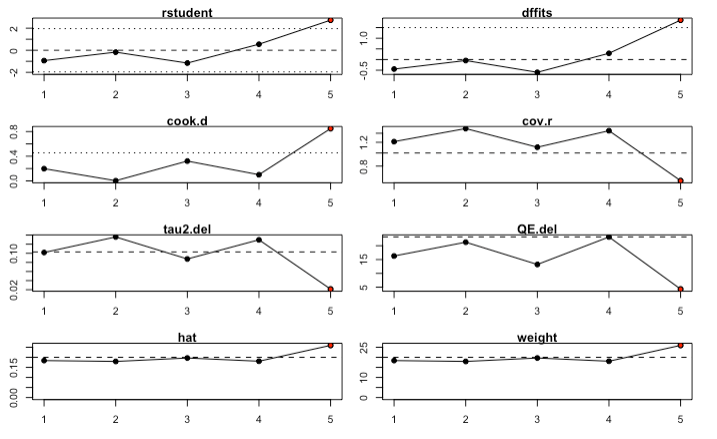


The study highlighted in red is identified as a potential outlier. Study 5 corresponds to Lang et al., 2021.^19^

**CVR outlier tests (HbA_1c_)**


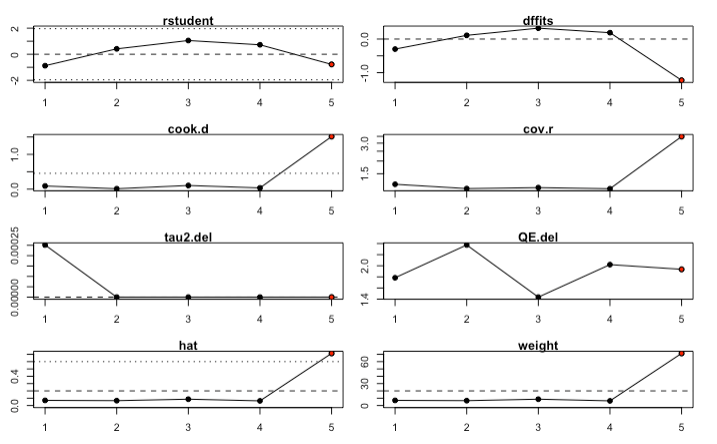


The study highlighted in red is identified as a potential outlier. Study 5 corresponds to Lang et al., 2021.^19^

**eAppendix 9: Total cholesterol results**

**SMD forest plot (total cholesterol)**

**
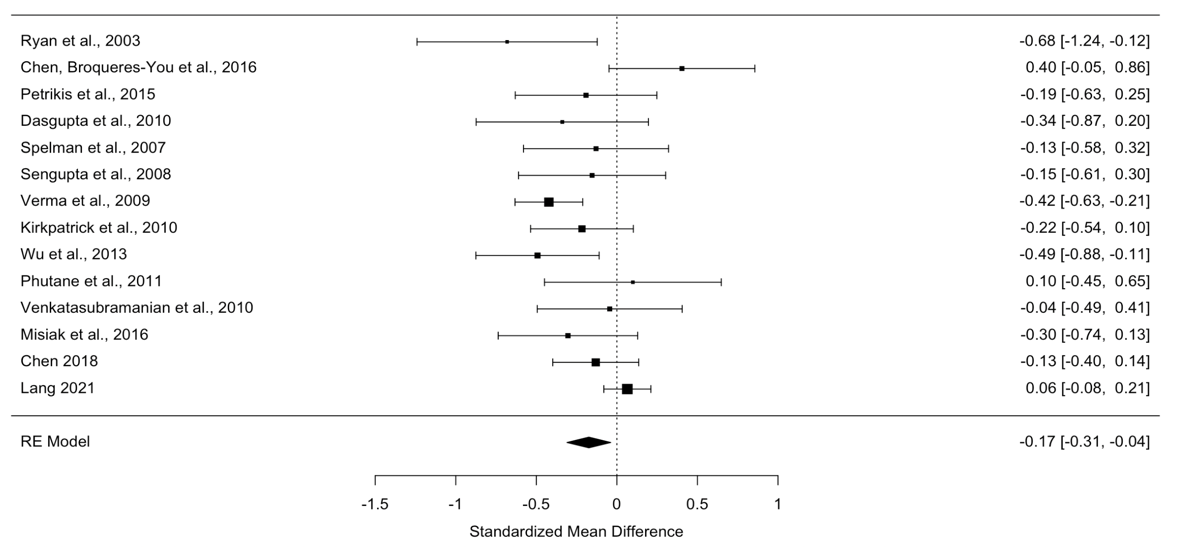
**

**CVR forest plot (total cholesterol)**

**
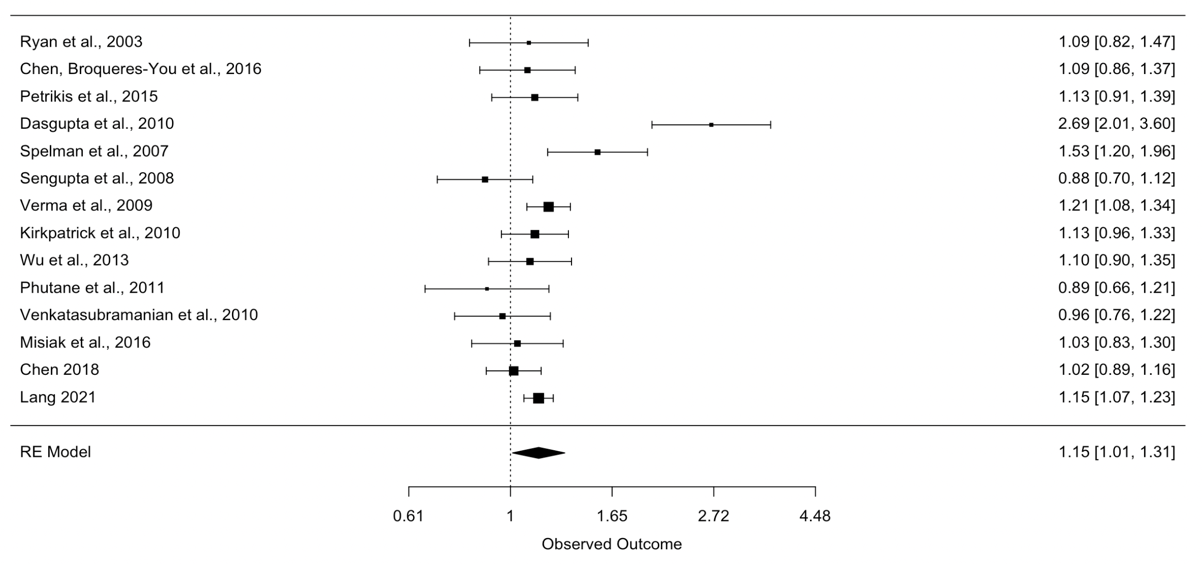
**

**Funnel plot (total cholesterol)**

**
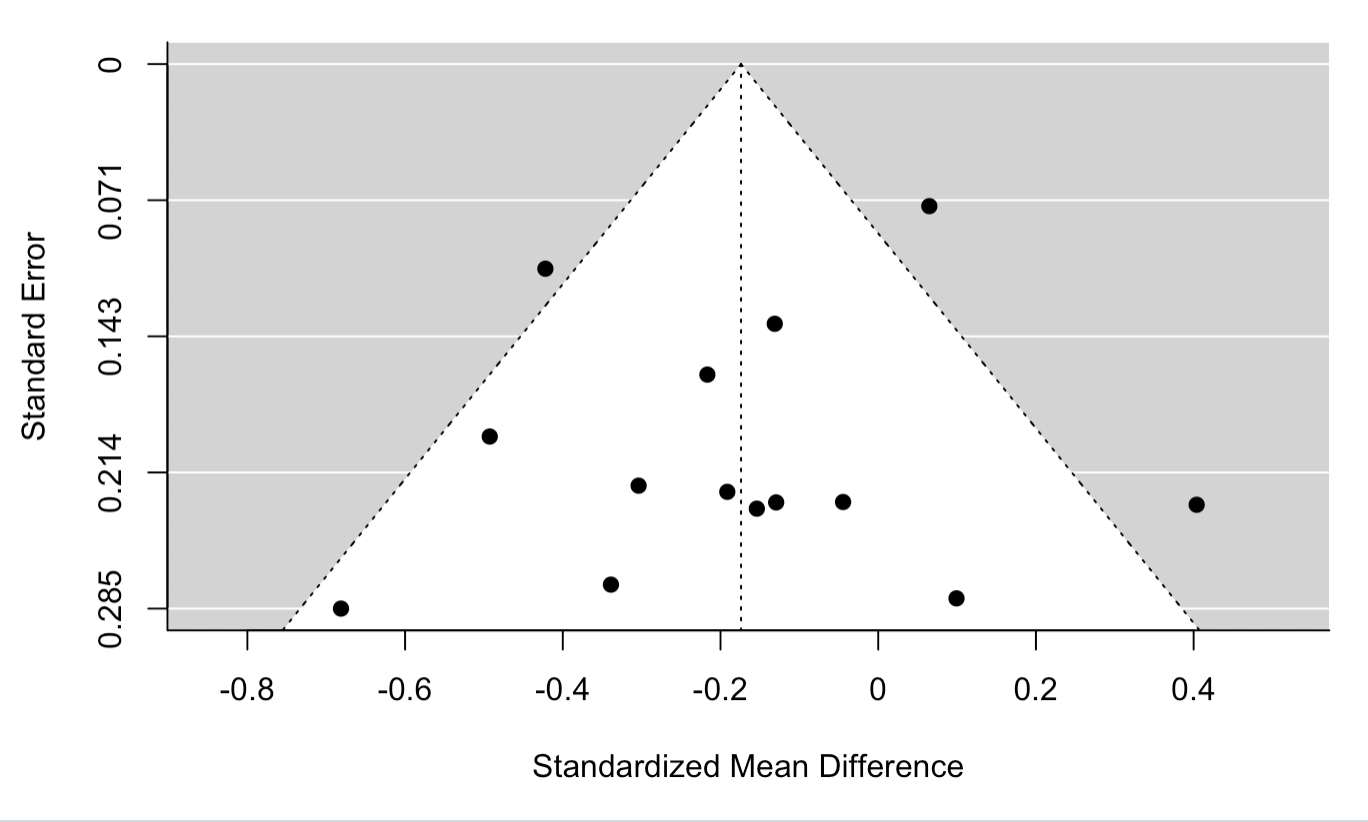
**

**SMD outlier analysis (total cholesterol)**

**
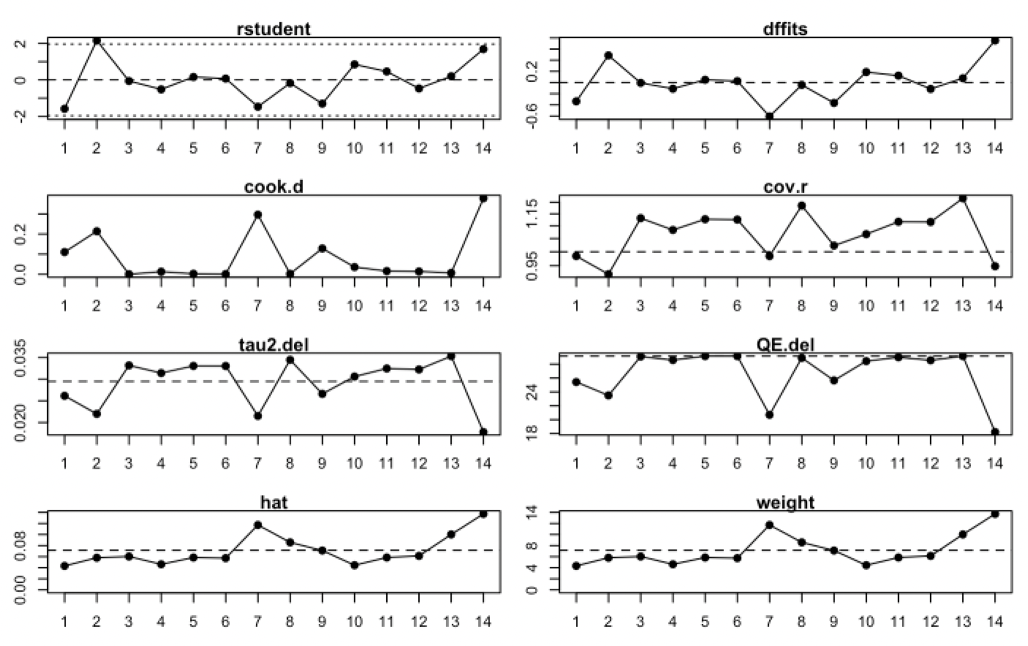
**

No outliers identified.

**CVR outlier analysis (total cholesterol)**

**
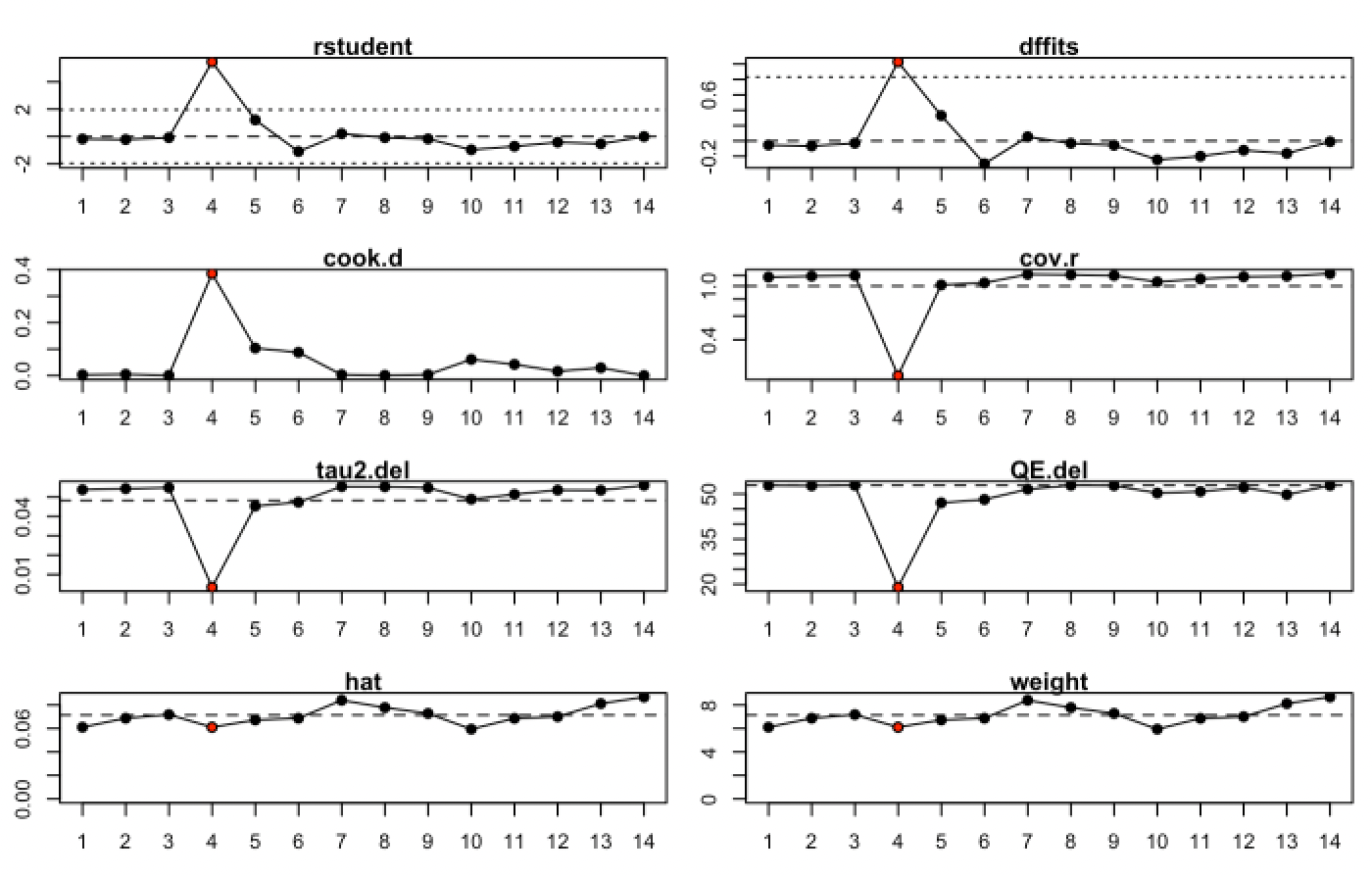
**

The study highlighted in red is identified as a potential outlier. Study 4 corresponds to Dasgupta et al., 2010.^13^

**eAppendix 10: LDL cholesterol results**

**SMD forest plot (LDL cholesterol)**

**
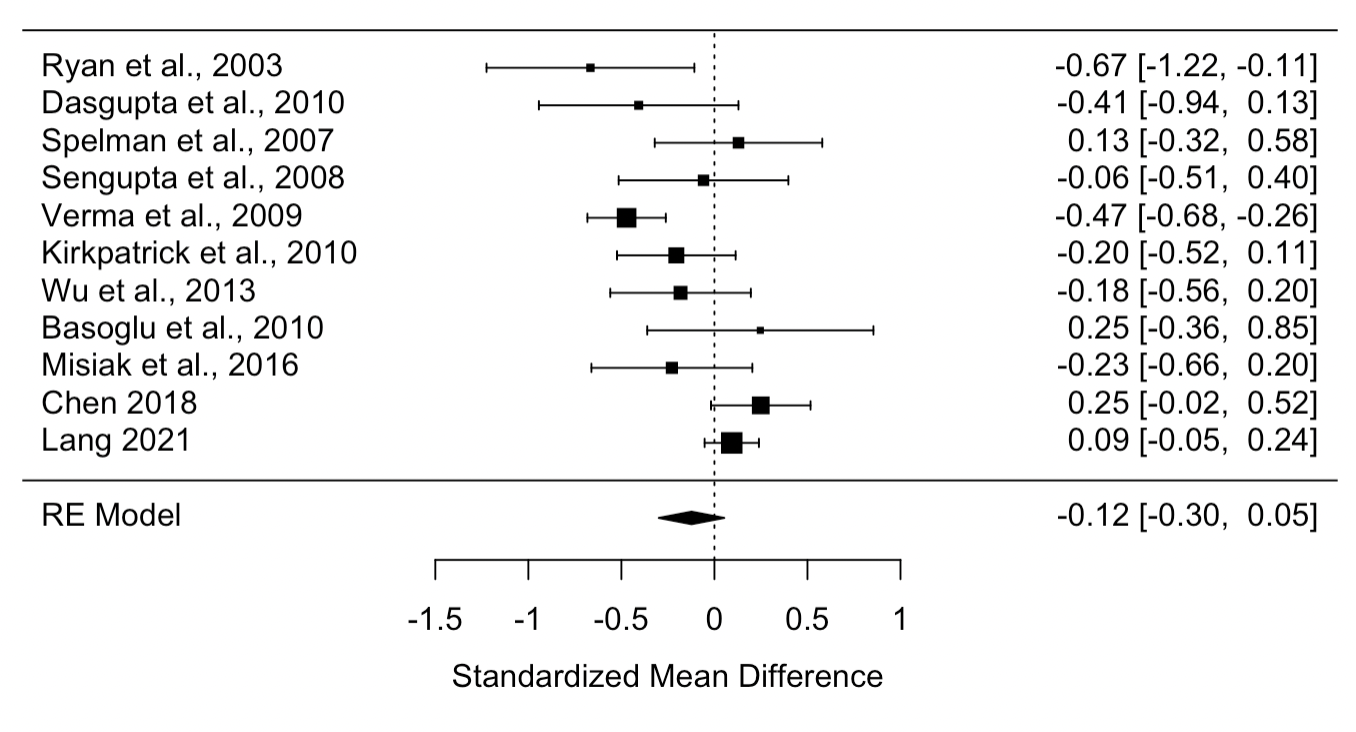
**

**CVR forest plot (LDL cholesterol)**

**
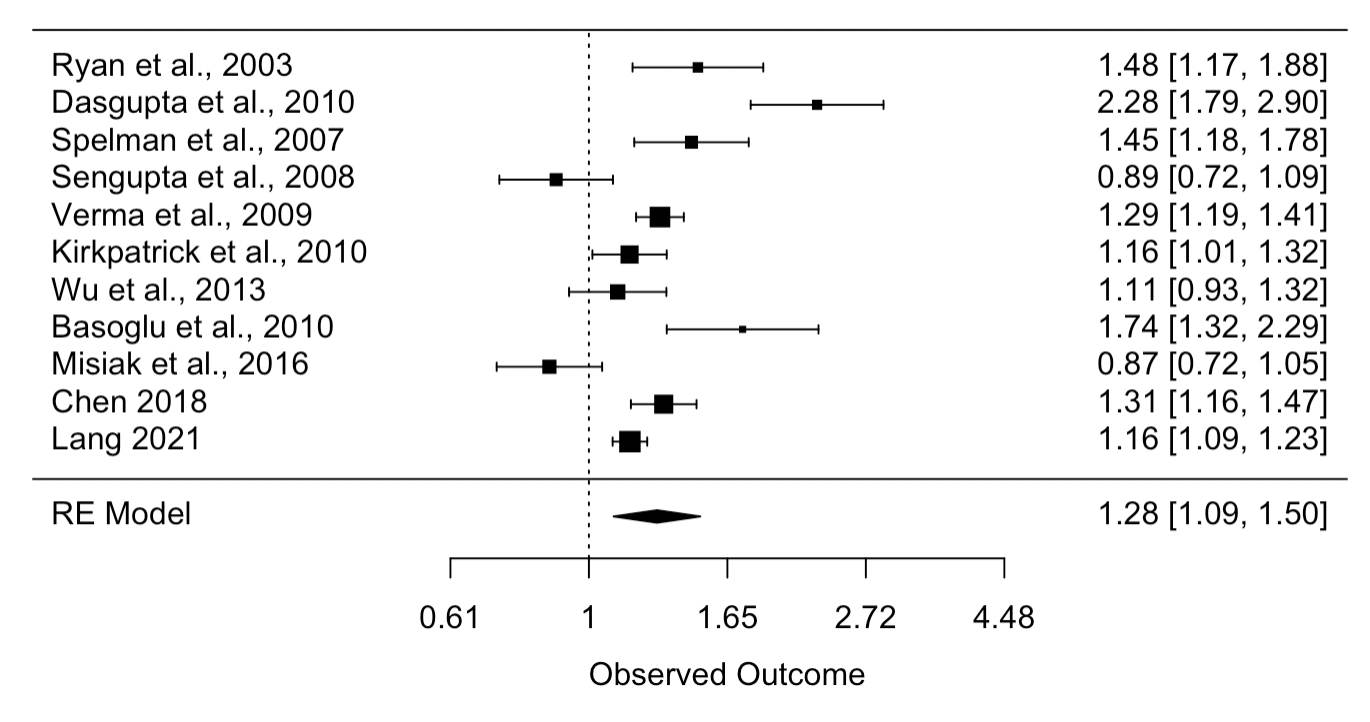
**

**Funnel plot (LDL cholesterol)**

**
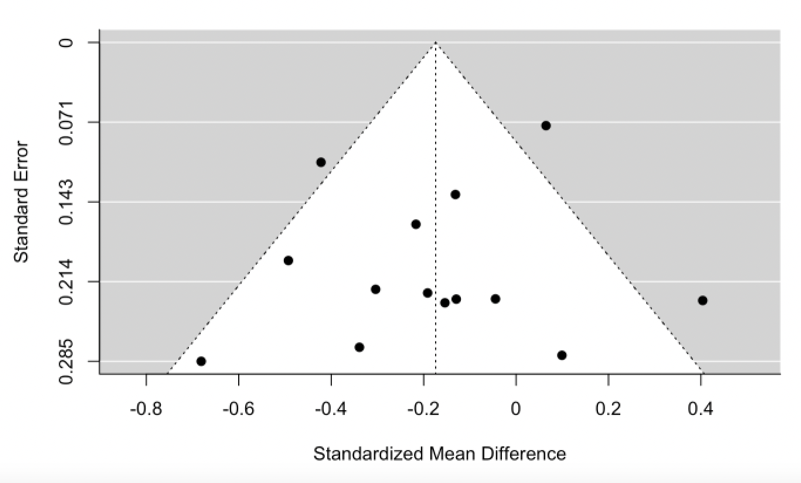
**

**SMD outlier analysis (LDL cholesterol)**

**
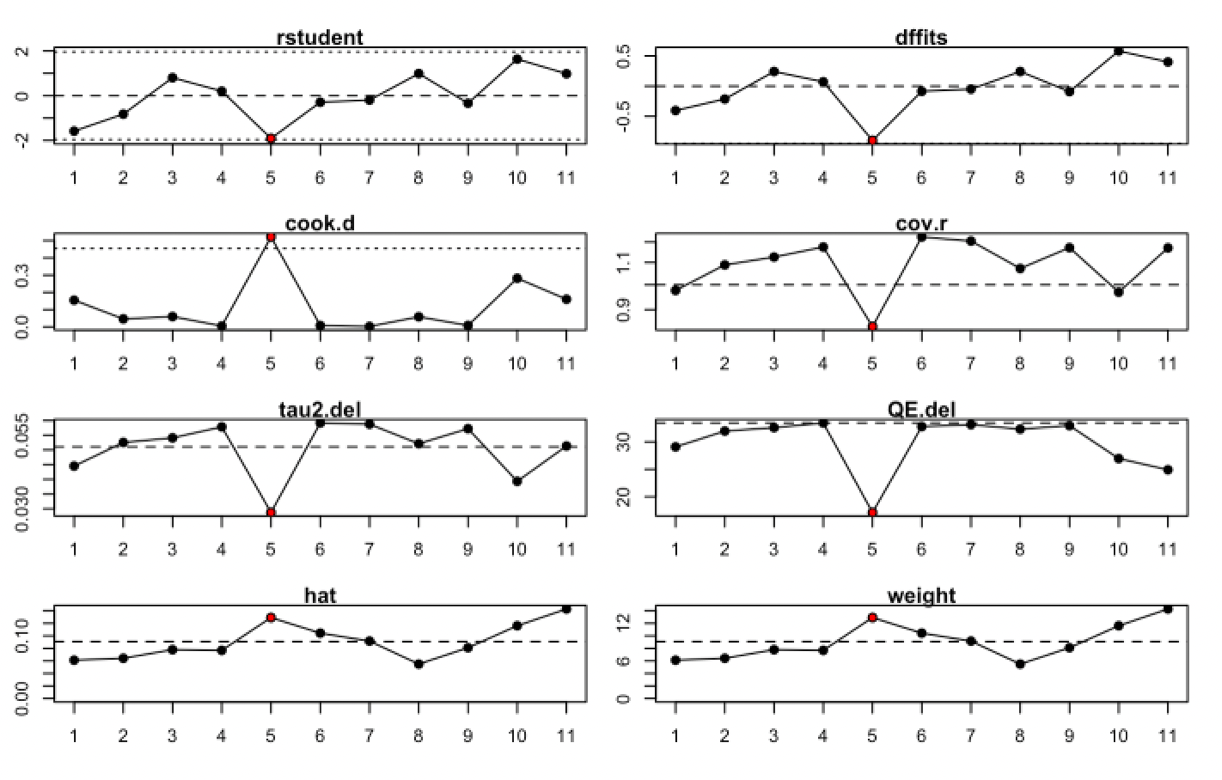
**

The study highlighted in red is identified as a potential outlier. Study 5 corresponds to Verma et al., 2010.^29^

**CVR outlier analysis (LDL cholesterol)**

**
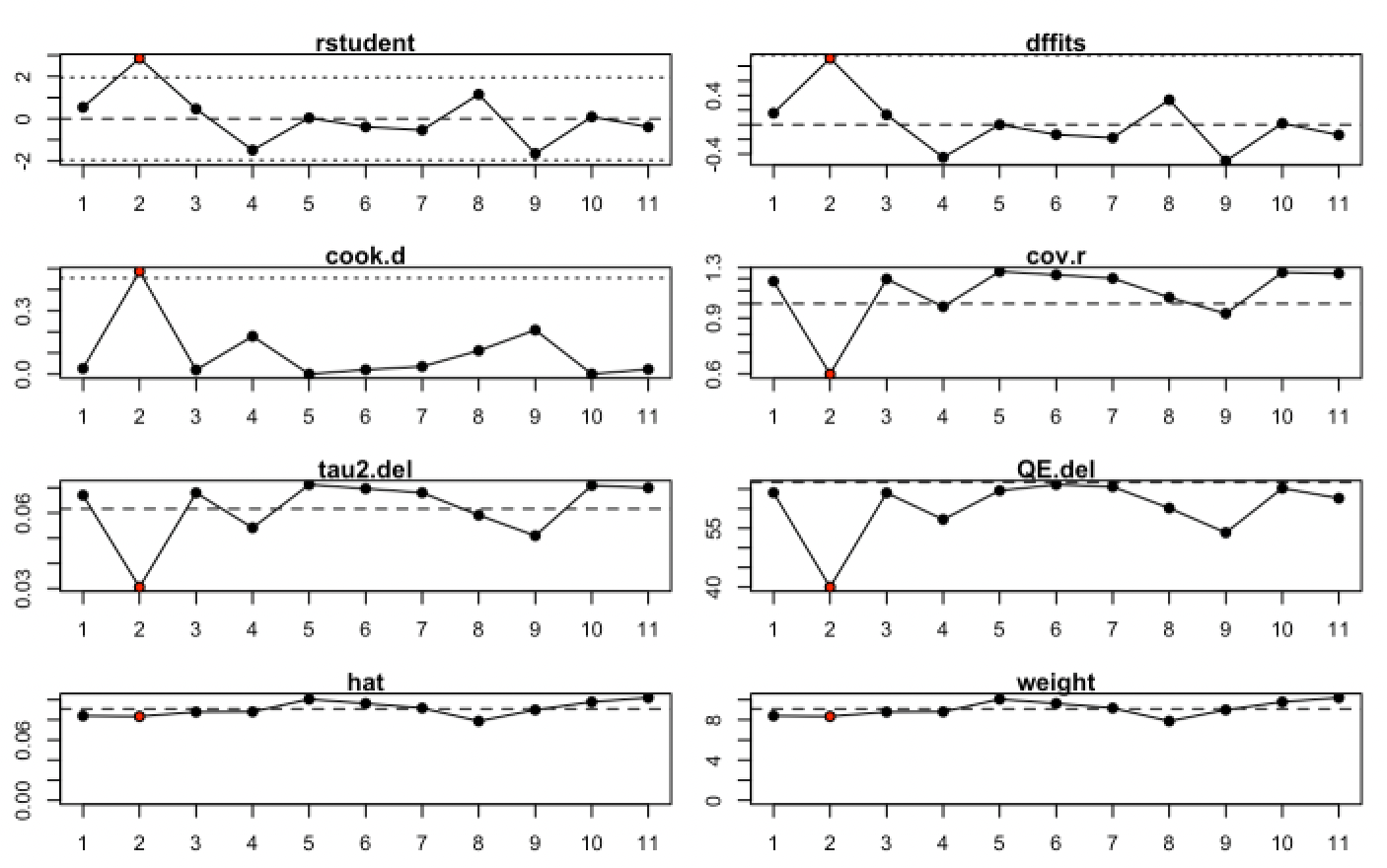
**

The study highlighted in red is identified as a potential outlier. Study 2 corresponds to Dasgupta et al., 2009.^13^

**eAppendix 11: HDL cholesterol results**

**SMD forest plot (HDL cholesterol)**

**
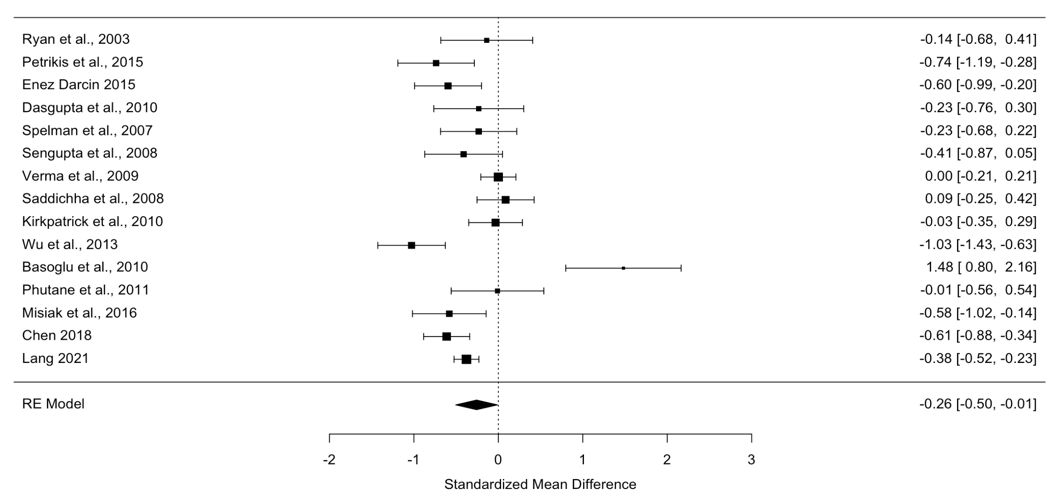
**

**CVR forest plot (HDL cholesterol**

**
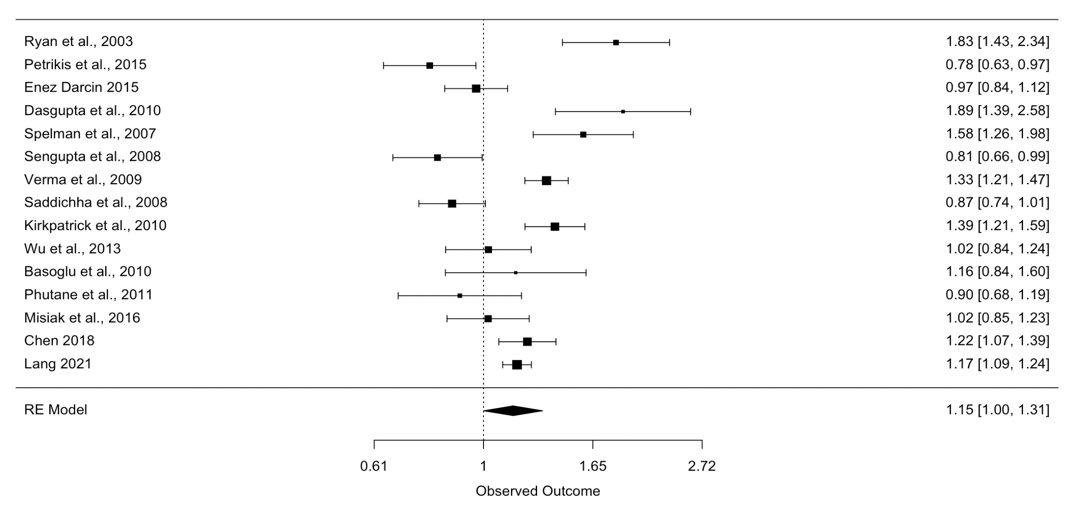
**

**Funnel plot (HDL cholesterol)**

**
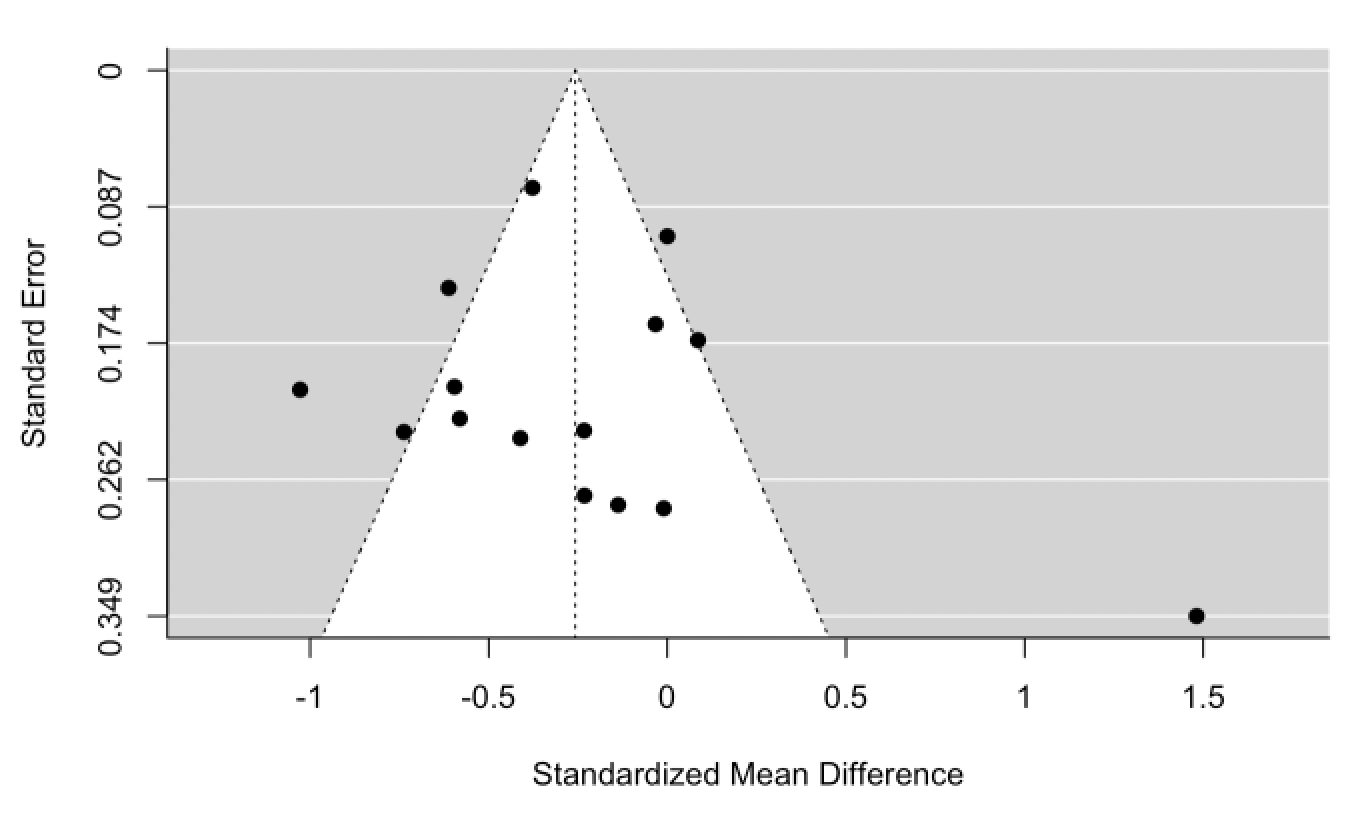
**

**SMD outlier analysis (HDL cholesterol)**

**
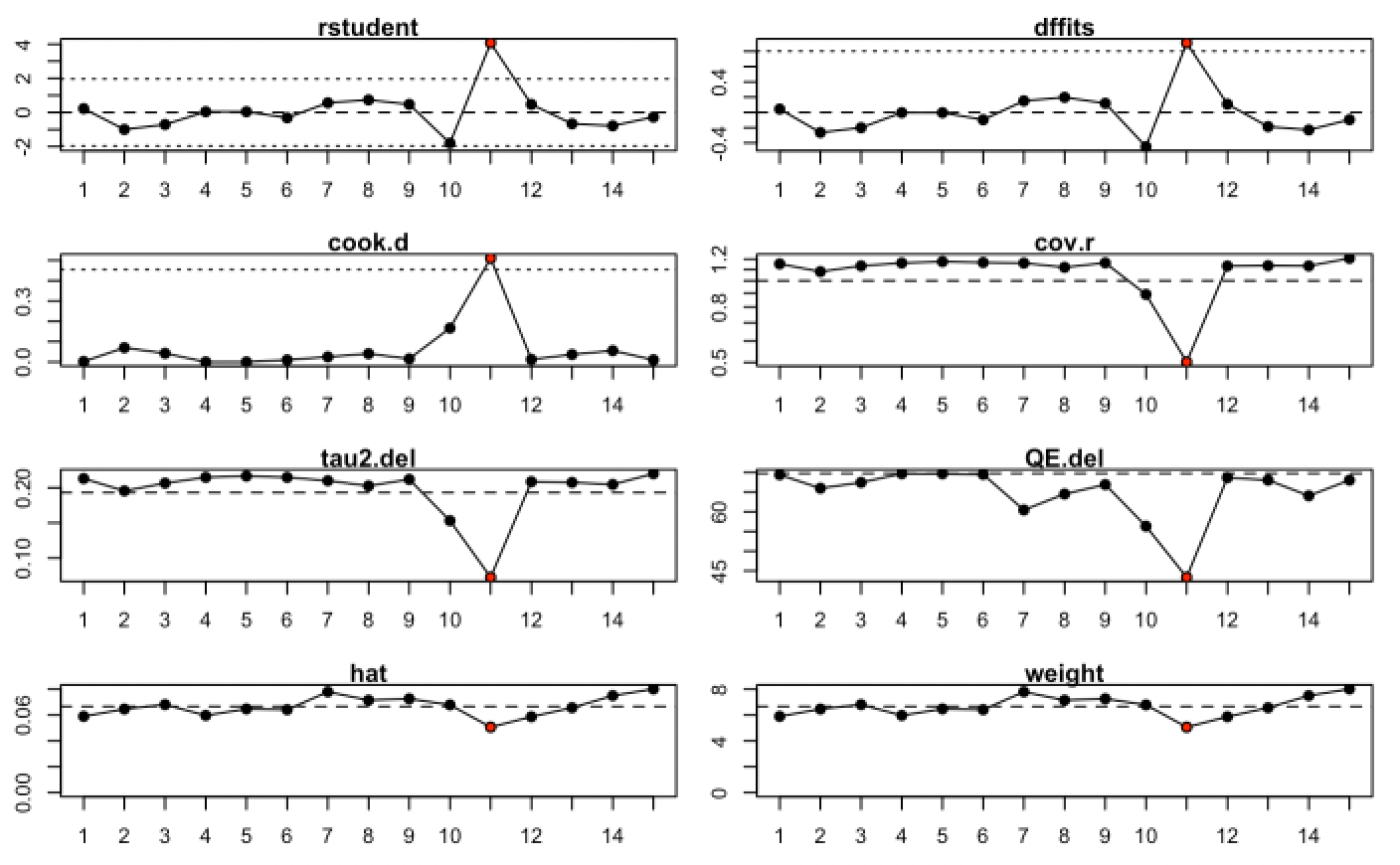
**

The study highlighted in red is identified as a potential outlier. Study 11 corresponds to Basoglu et al., 2010.^6^

**CVR outlier analysis (HDL cholesterol)**

**
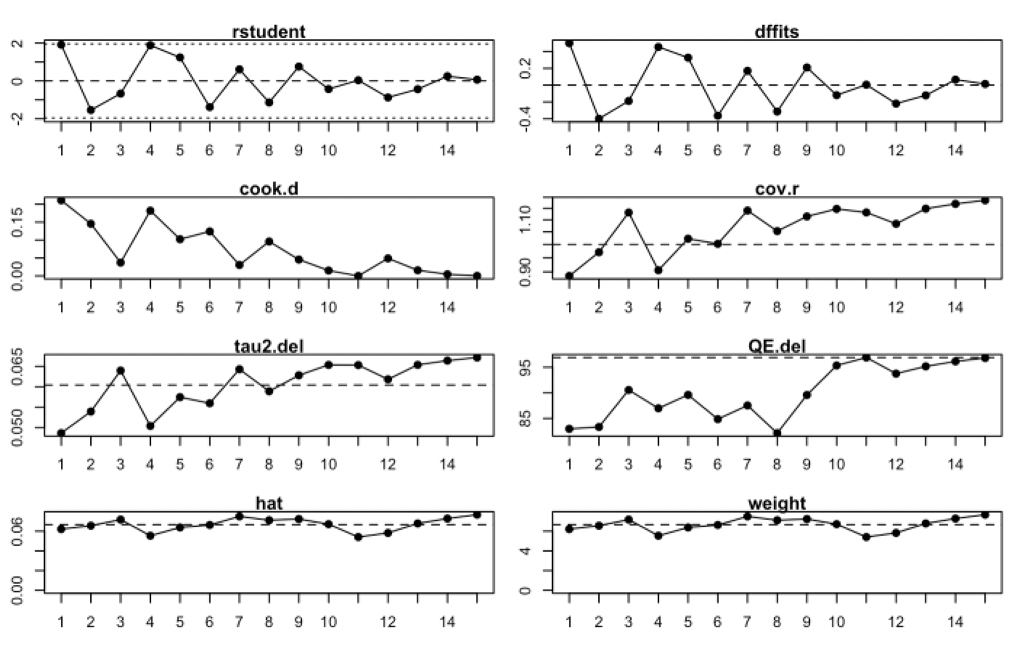
**

No outliers identified.

**eAppendix 12: Triglyceride results**

**SMD forest plot (triglycerides)**

**
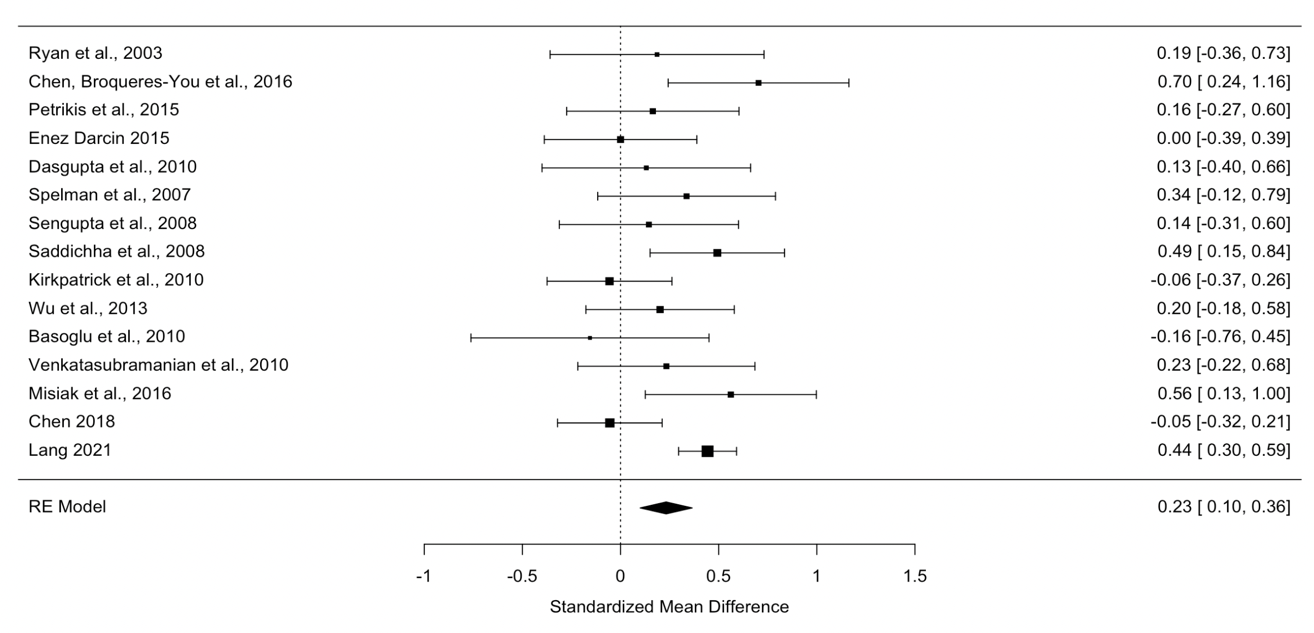
**

**CVR forest plot (triglycerides)**

**
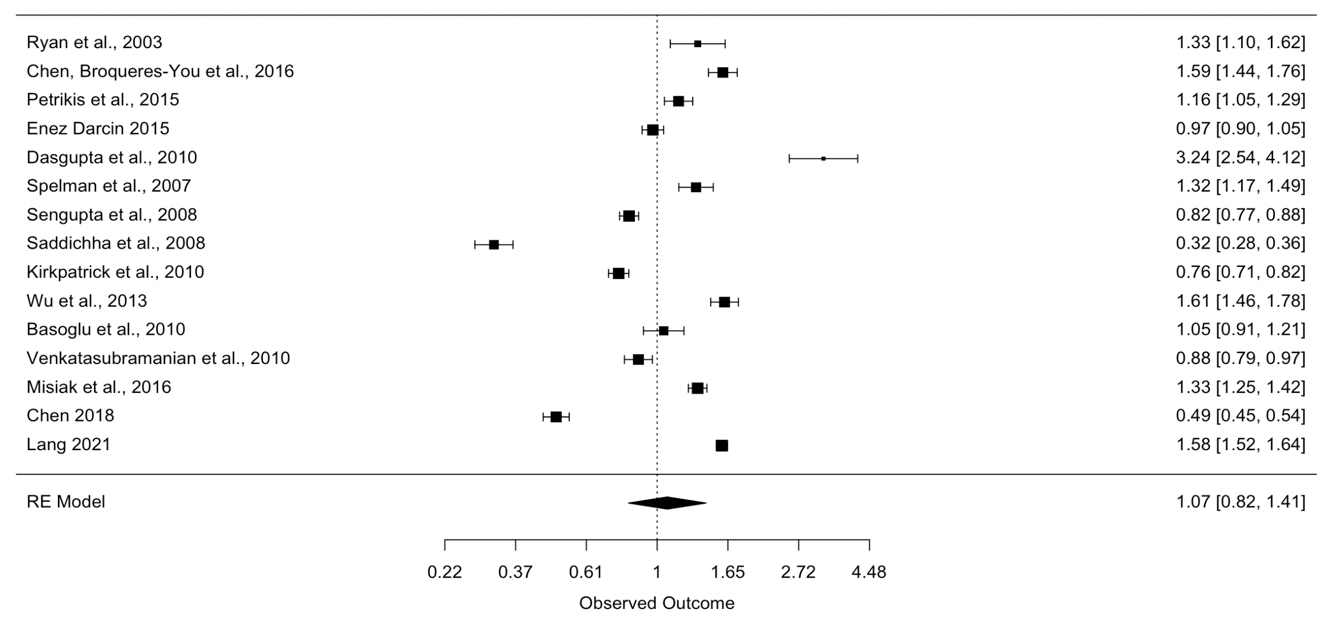
**

**Funnel plot (triglycerides)**

**
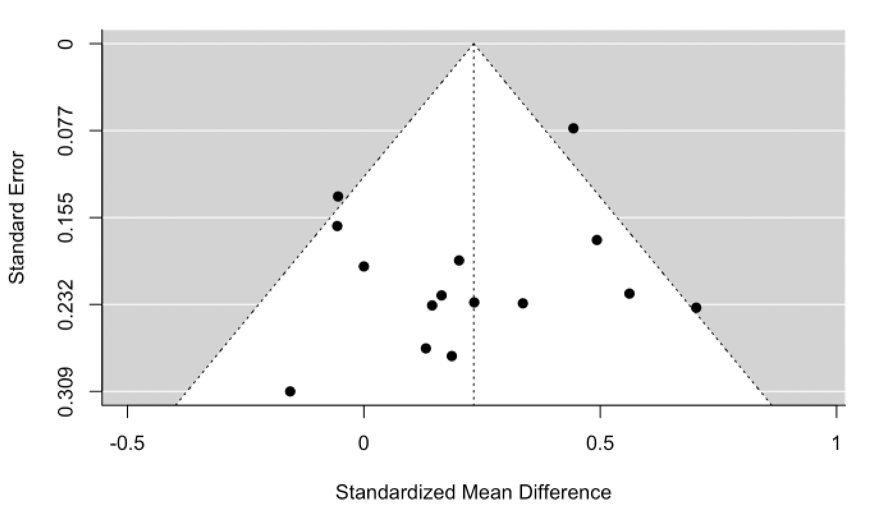
**

**SMD outlier analysis (triglycerides)**

**
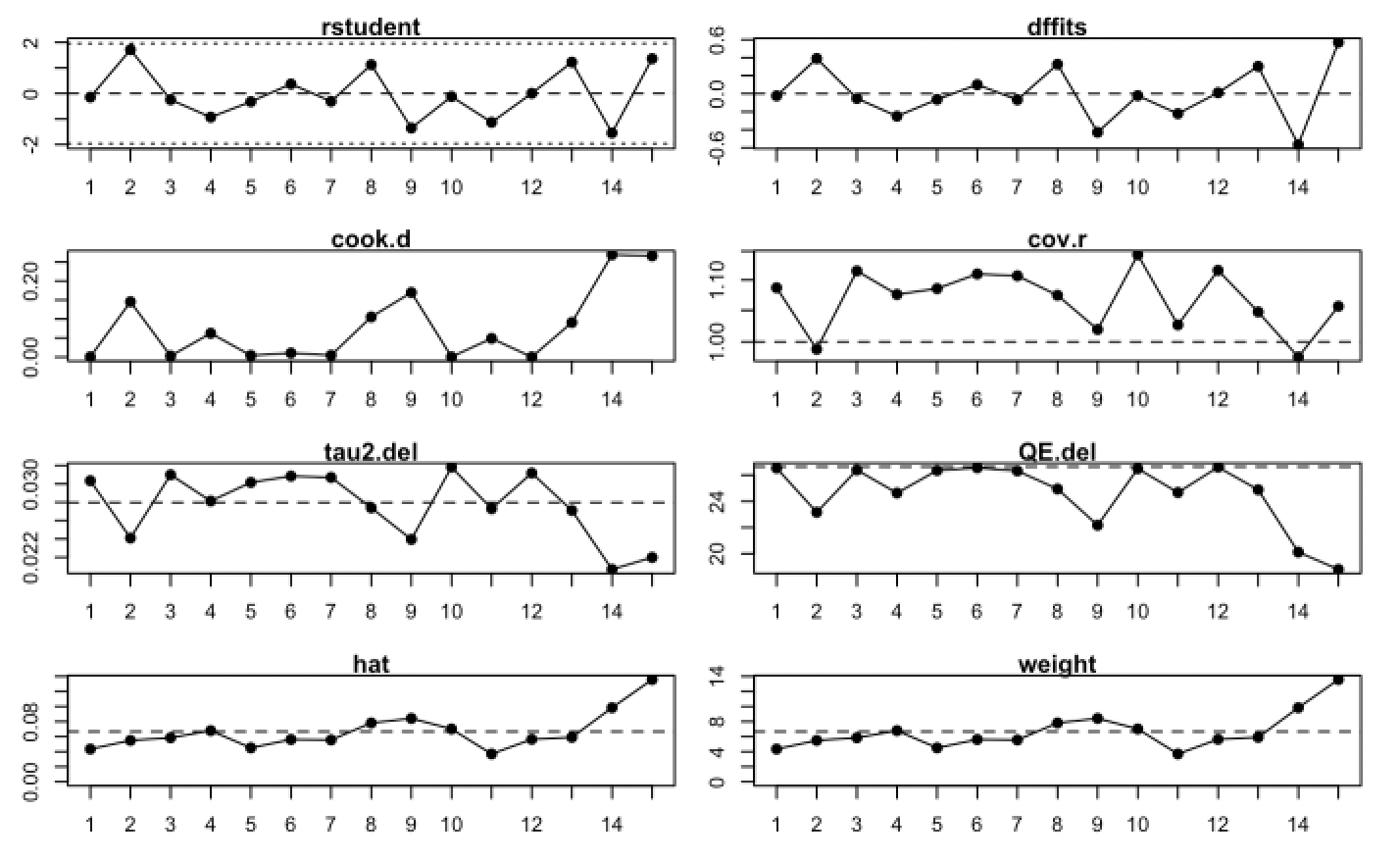
**

No outliers identified.

**CVR outlier analysis (triglycerides)**

**
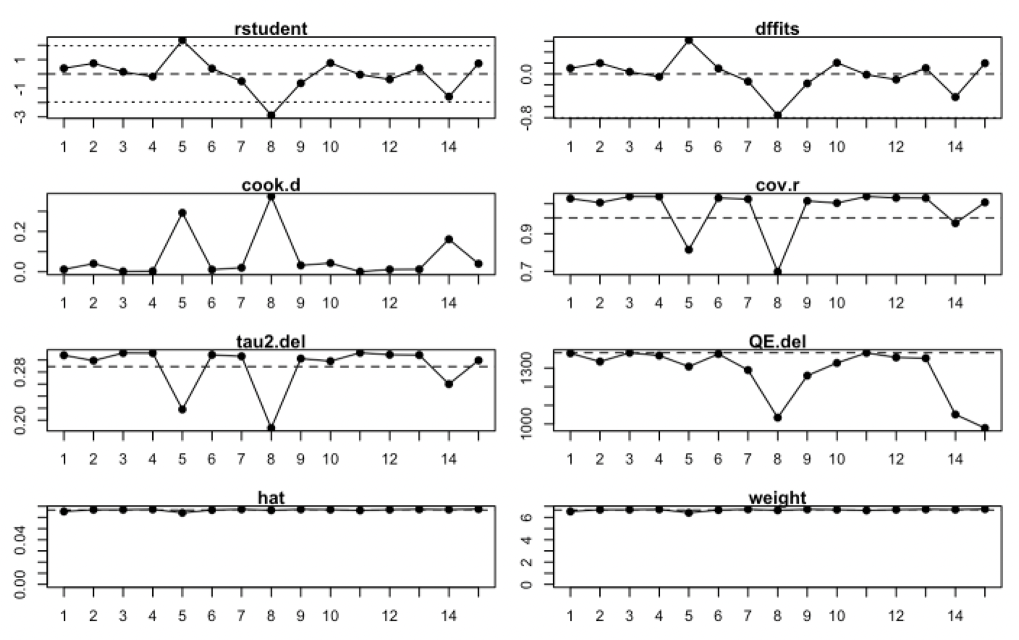
**

No outliers identified.

**References**

1. Pillinger T, Beck K, Gobjila C, Donocik JG, Jauhar S, Howes OD. Impaired glucose homeostasis in first-episode schizophrenia: A systematic review and meta-analysis. *JAMA Psychiatry* 2017; **74**(3)**:** 261-269.

2. Pillinger T, Beck K, Stubbs B, Howes OD. Cholesterol and triglyceride levels in first-episode psychosis: Systematic review and meta-analysis. *Brit J Psychiat* 2017; **211**(6)**:** 339-349.

3. Nakagawa S, Poulin R, Mengersen K, Reinhold K, Engqvist L, Lagisz M *et al.* Meta-analysis of variation: ecological and evolutionary applications and beyond. *Methods Ecol Evol* 2015; **6**(2)**:** 143-152.

4. Fronczak A, Fronczak P. Origins of Taylor's power law for fluctuation scaling in complex systems. *Phys Rev E Stat Nonlin Soft Matter Phys* 2010; **81**(6 Pt 2)**:** 066112.

5. Arranz B, Rosel P, Ramirez N, Duenas R, Fernandez P, Sanchez JM *et al.* Insulin resistance and increased leptin concentrations in noncompliant schizophrenia patients but not in antipsychotic-naive first-episode schizophrenia patients. *J Clin Psychiatry* 2004; **65**(10)**:** 1335-1342.

6. Basoglu C, Oner O, Gunes C, Semiz UB, Ates AM, Algul A *et al.* Plasma orexin A, ghrelin, cholecystokinin, visfatin, leptin and agouti-related protein levels during 6-week olanzapine treatment in first-episode male patients with psychosis. *Int Clin Psychopharmacol* 2010; **25**(3)**:** 165-171.

7. Cai HL, Li HD, Yan XZ, Sun B, Zhang Q, Yan M *et al.* Metabolomic analysis of biochemical changes in the plasma and urine of first-episode neuroleptic-naive schizophrenia patients after treatment with risperidone. *J Proteome Res* 2012; **11**(8)**:** 4338-4350.

8. Chen S, Broqueres-You D, Yang G, Wang Z, Li Y, Wang N *et al.* Relationship between insulin resistance, dyslipidaemia and positive symptom in Chinese antipsychotic-naive first-episode patients with schizophrenia. *Psychiatry Res* 2013; **210**(3)**:** 825-829.

9. Chen DC, Du XD, Yin GZ, Yang KB, Nie Y, Wang N *et al.* Impaired glucose tolerance in first-episode drug-naive patients with schizophrenia: relationships with clinical phenotypes and cognitive deficits. *Psychol Med* 2016**:** 1-12.

10. Chen S, Broqueres-You D, Yang G, Wang Z, Li Y, Yang F *et al.* Male sex may be associated with higher metabolic risk in first-episode schizophrenia patients: A preliminary study. *Asian J Psychiatr* 2016; **21:** 25-30.

11. Chen J, Tan L, Long Z, Wang L, Hu L, Yang D. Drug-naive patients with schizophrenia have metabolic disorders that are not associated with polymorphisms in the LEP (-2548G/A) and 5-HTR2C (-759C/T) genes. *International Journal of Clinical & Experimental Pathology* 2018; **11**(12)**:** 5969-5980.

12. Cohn TA, Remington G, Zipursky RB, Azad A, Connolly P, Wolever TM. Insulin resistance and adiponectin levels in drug-free patients with schizophrenia: A preliminary report. *Can J Psychiatry* 2006; **51**(6)**:** 382-386.

13. Dasgupta A, Singh OP, Rout JK, Saha T, Mandal S. Insulin resistance and metabolic profile in antipsychotic naive schizophrenia patients. *Prog Neuropsychopharmacol Biol Psychiatry* 2010; **34**(7)**:** 1202-1207.

14. Enez Darcin A, Yalcin Cavus S, Dilbaz N, Kaya H, Dogan E. Metabolic syndrome in drug-naive and drug-free patients with schizophrenia and in their siblings. *Schizophr Res* 2015; **166**(1-3)**:** 201-206.

15. Fernandez-Egea E, Bernardo M, Donner T, Conget I, Parellada E, Justicia A *et al.* Metabolic profile of antipsychotic-naive individuals with non-affective psychosis. *Br J Psychiatry* 2009; **194**(5)**:** 434-438.

16. Garcia-Rizo C, Kirkpatrick B, Fernandez-Egea E, Oliveira C, Bernardo M. Abnormal glycemic homeostasis at the onset of serious mental illnesses: A common pathway. *Psychoneuroendocrinology* 2016; **67:** 70-75.

17. Kavzoglu SO, Hariri AG. Intracellular Adhesion Molecule (ICAM-1), Vascular Cell Adhesion Molecule (VCAM-1) and E-Selectin Levels in First Episode Schizophrenic Patients. *Klin Psikofarmakol B* 2013; **23**(3)**:** 205-214.

18. Kirkpatrick B, Garcia-Rizo C, Tang K, Fernandez-Egea E, Bernardo M. Cholesterol and triglycerides in antipsychotic-naive patients with nonaffective psychosis. *Psychiatry Res* 2010; **178**(3)**:** 559-561.

19. Lang X, Zhou Y, Zhao L, Gu Y, Wu X, Zhao Y *et al.* Differences in patterns of metabolic abnormality and metabolic syndrome between early-onset and adult-onset first-episode drug-naive schizophrenia patients. *Psychoneuroendocrinology* 2021; **132 (no pagination)**.

20. Misiak B, Laczmanski L, Sloka NK, Szmida E, Piotrowski P, Loska O *et al.* Metabolic dysregulation in first-episode schizophrenia patients with respect to genetic variation in one-carbon metabolism. *Psychiatry Res* 2016; **238:** 60-67.

21. Petrikis P, Tigas S, Tzallas AT, Papadopoulos I, Skapinakis P, Mavreas V. Parameters of glucose and lipid metabolism at the fasted state in drug-naive first-episode patients with psychosis: Evidence for insulin resistance. *Psychiatry Res* 2015; **229**(3)**:** 901-904.

22. Phutane VH, Tek C, Chwastiak L, Ratliff JC, Ozyuksel B, Woods SW *et al.* Cardiovascular risk in a first-episode psychosis sample: a 'critical period' for prevention? *Schizophr Res* 2011; **127**(1-3)**:** 257-261.

23. Ryan MC, Collins P, Thakore JH. Impaired fasting glucose tolerance in first-episode, drug-naive patients with schizophrenia. *Am J Psychiatry* 2003; **160**(2)**:** 284-289.

24. Saddichha S, Manjunatha N, Ameen S, Akhtar S. Metabolic syndrome in first episode schizophrenia - a randomized double-blind controlled, short-term prospective study. *Schizophr Res* 2008; **101**(1-3)**:** 266-272.

25. Sengupta S, Parrilla-Escobar MA, Klink R, Fathalli F, Ying Kin N, Stip E *et al.* Are metabolic indices different between drug-naive first-episode psychosis patients and healthy controls? *Schizophr Res* 2008; **102**(1-3)**:** 329-336.

26. Spelman LM, Walsh PI, Sharifi N, Collins P, Thakore JH. Impaired glucose tolerance in first-episode drug-naive patients with schizophrenia. *Diabet Med* 2007; **24**(5)**:** 481-485.

27. Sun HQ, Li SX, Chen FB, Zhang Y, Li P, Jin M *et al.* Diurnal neurobiological alterations after exposure to clozapine in first-episode schizophrenia patients. *Psychoneuroendocrinology* 2016; **64:** 108-116.

28. Tao Q, Miao Y, Li H, Yuan X, Huang X, Wang Y *et al.* Insulin Resistance and Oxidative Stress: In Relation to Cognitive Function and Psychopathology in Drug-Naive, First-Episode Drug-Free Schizophrenia. *Frontiers in Psychiatry* 2020; **11 (no pagination)**.

29. Verma SK, Subramaniam M, Liew A, Poon LY. Metabolic risk factors in drug-naive patients with first-episode psychosis. *J Clin Psychiatry* 2009; **70**(7)**:** 997-1000.

30. Venkatasubramanian G, Chittiprol S, Neelakantachar N, Naveen MN, Thirthall J, Gangadhar BN *et al.* Insulin and insulin-like growth factor-1 abnormalities in antipsychotic-naive schizophrenia. *Am J Psychiatry* 2007; **164**(10)**:** 1557-1560.

31. Wani RA, Dar MA, Margoob MA, Rather YH, Haq I, Shah MS. Diabetes mellitus and impaired glucose tolerance in patients with schizophrenia, before and after antipsychotic treatment. *J Neurosci Rural Pract* 2015; **6**(1)**:** 17-22.

32. Wu X, Huang Z, Wu R, Zhong Z, Wei Q, Wang H *et al.* The comparison of glycometabolism parameters and lipid profiles between drug-naive, first-episode schizophrenia patients and healthy controls. *Schizophr Res* 2013; **150**(1)**:** 157-162.
